# External validation of the Leiden Thrombosis Recurrence Risk Prediction models (L-TRRiP) for the prediction of recurrence after a first venous thrombosis in the Heart and Vascular Health study

**DOI:** 10.1016/j.rpth.2024.102610

**Published:** 2024-10-29

**Authors:** J. Louise I. Burggraaf-van Delft, Kerri L. Wiggins, Nienke van Rein, Saskia le Cessie, Nicholas L. Smith, Suzanne C. Cannegieter

**Affiliations:** 1 Department of Clinical Epidemiology, Leiden University Medical Center, Leiden, the Netherlands; 2 Department of Medicine, University of Washington, Seattle, Washington, USA; 3 Department of Clinical Pharmacy and Toxicology, Leiden University Medical Center, Leiden, the Netherlands; 4 Department of Biomedical Data Sciences, Leiden University Medical Center, Leiden, the Netherlands; 5 Department of Epidemiology, University of Washington, Seattle, Washington, USA; 6 Kaiser Permanente Washington Health Research Institute, Seattle, Washington, USA; 7 Department of Veterans Affairs Office of Research and Development, Seattle Epidemiologic Research and Information Center, Seattle, Washington, USA; 8 Department of Medicine – Thrombosis and Hemostasis, Leiden University Medical Center, Leiden, the Netherlands

**Keywords:** anticoagulants, clinical decision rules, prognosis, validation study, venous thromboembolism

## Abstract

**Background:**

Long-term outcome after a first venous thromboembolism (VTE) might be optimized by tailoring anticoagulant treatment duration on individual risks of recurrence and major bleeding. The L-TRRiP models (A–D) were previously developed in data from the Dutch Multiple Environment and Genetic Assessment of Risk Factors for Venous thrombosis study to predict VTE recurrence.

**Objectives:**

We aimed to externally validate models C and D using data from the United States Heart and Vascular Health (HVH) study.

**Methods:**

Data from participants with a first VTE who discontinued initial anticoagulant therapy were used to determine model performance. Missing data were imputed, and results were pooled according to Rubin’s rules. To determine discrimination, Harrell’s C-statistic was calculated. To assess calibration, the observed/expected (O/E) ratio was estimated, and calibration plots were created, in which we accounted for the competing risk of death. A stratified analysis based on age <70 or >70 years was performed.

**Results:**

Of 1430 participants from the HVH study, 187 experienced an unprovoked VTE recurrence during follow-up. The C-statistics of L-TRRIP models C and D were 0.62 (95% CI, 0.56-0.67) and 0.61 (95% CI, 0.55-0.67), respectively. The O/E ratio (1.00; 95% CI, 0.84-1.17 and 1.09; 95% CI, 0.91-1.27, respectively) and calibration plots indicated good calibration. The discrimination was similar between participants <70 or >70 years, whereas overall calibration was lower in participants <70 years.

**Conclusion:**

The L-TRRiP models showed moderate discrimination and good calibration in a different population and can be used to guide clinical decision making. To assess the added value in daily clinical practice, a management study is needed.

## Essentials

• Models to predict recurrent thrombosis might help to determine anticoagulant therapy duration.
• The performance of the L-TRRiP model was assessed using the Heart and Vascular Health study.
• The predicted recurrent thrombosis risk corresponded to the actual recurrence risk.
• The model might support clinical decisions but should be evaluated during implementation.

## 1 Introduction

After a first venous thromboembolism (VTE), the risk of a recurrent event is 3% to 15% within 5 years for patients with a provoked VTE and 25% for patients with an unprovoked VTE [[1], [2], [3]]. The risk of recurrent VTE can be reduced by prolonged anticoagulant therapy, but this comes at the cost of increased risk for major bleeding [4]. Ideally, treatment duration is based on the individual trade-off between benefits and harms, ie, the reduction in VTE recurrence risk vs the increased risk of bleeding. Estimating these risks accurately for an individual patient can be challenging, but prediction models have been developed to assist. However, it is essential that such models are sufficiently validated (internally and externally) before they can be implemented safely in clinical practice, preferably in different settings [[5], [6], [7]].

The L-TRRiP models (A-D) were developed to predict the absolute risk of an unprovoked recurrent VTE within 2 years after discontinuation of initial anticoagulant therapy in all patients with a first VTE without malignancy. The models were developed using data from the Multiple Environment and Genetic Assessment of Risk Factors for Venous thrombosis (MEGA) follow-up study, including 3750 patients of primarily European ancestry who experienced 507 recurrent events in 19.201 person-years [8,9]. Based on model performance and convenience of the included predictors, which can all be measured during anticoagulant treatment, model C (including clinical and genetic factors) is considered the most optimal model, as described previously [8].

The internal validation showed a C-statistic of 0.70 (95% CI, 0.68-0.73) for model C and good calibration. Upon external validation using data from the Tromsø study (n = 578, 73 recurrent events), the C-statistic of model C was 0.64 (95% CI, 0.62-0.66), and predicted risks were slightly overestimated [[8], [9], [10]].

However, since this external validation was limited by the small population, the use of different definitions for 2 predictor variables, and the fact that it did not account for the competing risk of death, we aimed to perform an additional validation study.

The aim of this study was to assess the predictive performance of the most optimal L-TRRiP model, L-TRRiP model C, using data from HVH study participants who suffered a first VTE, including a subgroup analysis in participants aged ≥70 and sensitivity analysis accounting for the competing risk of death. In addition, we assessed the performance of L-TRRiP model D as well since this model only includes clinical variables and might therefore be a useful alternative.

## 2 Methods

### 2.1 Population

The HVH study is a population-based case-control study that includes individuals who experienced cardiovascular events, including a first VTE, and also included genetic analyses and performed a follow-up of the VTE cases for VTE recurrence. Hence, all data required for validation of L-TRRiP models C and D are available within the HVH data. Details of the study design have been published previously [[11], [12], [13], [14]]. In short, potential VTE participants were identified from Group Health Cooperative, now Kaiser Permanente Washington, a community-based health care cooperative, using *International Classification of Diseases* codes and prescriptions for low molecular weight heparin. Women aged 18 to 89 years with a VTE occurring between 1995 and 2010 and men aged 30 to 89 years with a VTE occurring between 2002 and 2010 were eligible for possible study inclusion [11,12]. For all participants, baseline information, including demographic information, risk factors for VTE and data of the index event, was collected from the medical records, diagnosis codes, and pharmacy records and reviewed by trained abstractors to confirm study eligibility and the occurrence of a VTE event [11,12]. Eligible participants known to be alive and capable of completing a telephone interview were contacted by phone within 1 to 2 years after the index event and asked to provide a blood sample. Genotyping was performed in participants for whom white blood cell samples were available. Participants did not receive the results of genotyping. Participants with a first VTE between 2002 to 2010 were included in the follow-up, and their medical records underwent a second abstractor review to identify recurrent VTEs. These participants were followed until they experienced a recurrent event, until the last day of health care follow-up documentation in the health record, disenrollment from the health care cooperative, death, or the end of the study (December 2014). The HVH study was approved by the institutional review board of Group Health Cooperative/Kaiser Permanente Washington; informed consent was provided by participants who were contacted for blood sampling and waived by the institutional review board for those who could not be contacted.

For the current study, we included individuals with a first VTE who were included in the follow-up study for VTE recurrence. We excluded participants who did not receive initial anticoagulant therapy, who had an upper extremity deep venous thrombosis (DVT) as the index event, who had a malignancy <5 years prior to the index event, who did not have any follow-up, or who had a VTE recurrence or end of follow-up before discontinuation of initial anticoagulant therapy. These exclusion criteria align with the MEGA study in which the L-TRRiP models were developed [8]. Since only cancer diagnosis <2 years prior to the index event were collected in the HVH data, we used linked data from the US Cancer Registry to identify participants with cancer diagnosis between 2 and 5 years prior to the index VTE.

### 2.2 Prediction models

Both L-TRRiP model C (clinical and genetic factors) and model D (clinical factors only) were validated. The clinical factors are male sex, type of first VTE, location of DVT (if applicable), previous surgery, pregnancy/puerperium, hormone use, plaster cast, immobility in bed at the hospital, and history of cardiovascular disease. The genetic factors included in model C are blood group (O vs non-O) and factor V Leiden mutation (absence vs homo- or heterozygous presence) (Table 1).

**Table 1:** L-TRRiP model C and D.

Factors	Model C	Model D
	Coefficient	Coefficient
Male sex	0.63	0.68
Type of first VTE		
PE	−0.61	−0.69
PE + DVT	0.32	0.31
Location of DVT		
Popliteal or distal DVT	−0.39	−0.49
Surgery^a^	−0.51	−0.52
Pregnancy/puerperium^a^	−1.49	−1.44
Hormone use^b^	−0.67	−0.62
Plaster cast^a^	−0.79	−0.83
Immobility in bed, in hospital^a^^,^^c^	−0.31	−0.34
History of cardiovascular disease^d^	−0.36	−0.37
Blood group non-O	0.24	−
Factor V Leiden mutation^e^	0.40	−
Baseline 2-year recurrence-free probability^f^	0.9235595	0.9019939

DVT, deep venous thrombosis; PE, pulmonary embolism; VTE, venous thromboembolism.

Table adapted from Timp et al. [9].

a Within 3 months prior to the index event.

b At the time of the index event.

c For ≥3 days.

d Including heart failure, angina pectoris, arterial insufficiency of the legs, and myocardial infarction.

e Heterozygous and homozygous.

f The baseline recurrence-free probability (S_0_) can be used to calculate the absolute 2-year predicted risk of recurrence using the following equation: risk of recurrence = 1 − S_0_ ∗∗ exp(prognostic score). The prognostic score is equal to beta1∗x1 + beta2∗x2 + beta 3∗x3…, where x1, x2, x3 represent the variables in the model and beta1, beta2, beta3, etc., represent the corresponding coefficients.

### 2.3 Predictors

Factor V Leiden and blood group were only available for participants in whom genotyping was performed. For the factors of immobilization in hospital, recent surgery, and history of cardiovascular disease, we used slightly different definitions compared with the original L-TRRiP model, based on data availability in the HVH study and using the most common clinical definition for these factors to reflect future use in clinical practice as much as possible. Sensitivity analyses assessed the impact of our chosen definitions for recent surgery and history of cardiovascular disease. For all other predictors we used the same definitions as in the development study. An overview of the exact predictor definitions is included in Supplementary Table 1.

### 2.4 Outcome

The outcome was unprovoked VTE recurrence after discontinuation of initial anticoagulant treatment; hence, follow-up for the current study started at the time of anticoagulant discontinuation. Recurrent VTE included recurrent pulmonary embolism (PE), DVT of the leg, or fatal VTE and was considered unprovoked in case there was no malignancy diagnosis within 6 months prior to the recurrence, no hormone use at the time of recurrence, and no hospitalization (including hospitalization for inpatient surgery) or pregnancy within 3 months prior to the recurrence. Participants with a recurrent VTE other than PE or lower extremity DVT (n = 1) were censored at the time of recurrence. Participants with a provoked recurrence were censored at the time of recurrence in the main analysis. In a sensitivity analysis, these provoked recurrences were added to the outcome definition.

### 2.5 Missing data

Missingness was most frequent for DVT location and genetic variables. Individuals who had a fatal first VTE, those who could not be contacted by phone, did not agree to a blood draw, or who had insufficient blood samples for white blood cell processing were not included in the genotyping. DVT location was missing more often in patients with concomitant PE and DVT. Based on these patterns of missingness, we assumed data were missing at random [15], and we performed multiple imputations creating 10 imputed datasets, for which results were pooled according to Rubin’s rules [16]. Detailed information on the percentage of missing data can be found in Table 2.

**Table 2:** Baseline characteristics of study population (at time of first VTE).

Variable	All patients	Missing data
	n (%) or mean (SD)	n (%)
Total number of participants	1430	
Age, y (SD)	63.2 (15.9)	-
Male sex	633 (44.3)	-
Type of first event		-
DVT only	701 (49.0)	
PE only	496 (34.7)	
PE + DVT	233 (16.3)	
Location of first DVT (if applicable)		92 (6.4)
Proximal DVT	436 (32.6)	
Popliteal or distal DVT^a^	406 (30.3)	
Provoked first event^b^^,^^c^	720 (50.3)	
Trauma	179 (12.6)	9 (0.6)
Surgery	394 (27.7)	6 (0.4)
Hospitalization >3 d	233 (16.3)	-
Plaster cast	86 (6.1)	10 (0.7)
Pregnancy, puerperium	15 (1.0)	-
Hormone use	184 (12.9)	-
Unprovoked first event	710 (49.7)	
History of cardiovascular disease	275 (19.2)	-
Non-O blood group	626 (67.0)	495 (34.6)
Factor V Leiden mutation	110 (11.8)	494 (34.5)

Continuous variables are denoted as mean (SD); categorical variables are denoted as number (%).

DVT, deep venous thrombosis; PE, pulmonary embolism; VTE, venous thromboembolism.

a Indicates DVT at the level of the vena poplitea or below.

b Index VTE was classified as provoked in case of major trauma, surgery, plaster cast, pregnancy, hospitalization >3 days, or hormone use in the 3 months prior to the index event.

c Total of all risk factors below exceeds the amount of provoking first events since multiple provoking risk factors could be present concomitantly.

### 2.6 Statistical analysis

Baseline characteristics were summarized as mean and SD for continuous variables and as percentage for categorical variables. For each individual, the probabilities of VTE recurrence at 2 years according to L-TRRiP models C and D were calculated (formulas are given in Table 1). To determine discrimination at the prediction horizon of 2 years, the C-index was calculated for the time range until 2 years of follow-up in each of the imputed datasets, and the results were pooled according to Rubin’s rules. The C-index was calculated using inverse probability of censoring weighting to adjust for censoring. Discriminative ability over the entire follow-up time was assessed by plotting the area under the receiving operator characteristic curve (AUC_t_) over time. To assess calibration, the predicted probability at 2 years (averaged over the 10 imputed datasets) was plotted against the observed probability of VTE recurrence at 2 years using pseudo-observations and locally estimated scatterplot smoothing. All analysis were corrected for the competing risk of death, using the methods and R code as described by van Geloven et al. [17]. To summarize overall calibration, the observed/expected (O/E) ratio at 2 years was calculated using the Aalen-Johansen estimator to account for the competing risk of death [17,18].

Furthermore, for model C, the model considered most optimal for future clinical use, we created a cumulative incidence curve of VTE recurrence accounting for the competing risk of death stratified by the predicted risk category (low, intermediate, or high, where an intermediate risk is defined as a predicted 2-year recurrence risk of 6%-14%). To visualize the range and overlap of predicted risks between provoked and unprovoked events, we created a histogram of the predicted risk scores stratified by provoked/unprovoked index event.

### 2.7 Stratified analysis and sensitivity analysis

Sensitivity analyses using altered predictor definitions and including provoked recurrences in the outcome definition were performed as described above (see Supplementary Table S1 for predictor definitions). Since the model was originally developed in a cohort of VTE patients aged <70 years, reflected by the lower mean age of patients in the MEGA study, we assessed model performance in patients aged <70 or >70 years separately to determine whether the model would still be reliable in elderly patients. In addition, we performed a sensitivity analysis without accounting for the competing risk of death as this was also not done during development and initial validation of the model [8]. Lastly, a sensitivity analysis excluding patients with distal DVT (below the level of the vena poplitea) was performed since the need and duration of anticoagulant therapy in these patients is not clearly established [19]. For this analysis, patients with DVT at the level of the vena poplitea were scored as distal DVT to calculate the predicted risk of VTE recurrence, according to the models’ predictor definitions.

Analyses were performed in Stata version 13 and in R version 4.3.1, using the packages cmprsk, dynpred, ggsurvfit, mice, lattice, pec, riskRegression, rsample, survival, tableone, and tidyverse. The R code used for the main analysis can be found in the Supplementary Material.

## 3 Results

### 3.1 Participant characteristics

**Figure 1:**
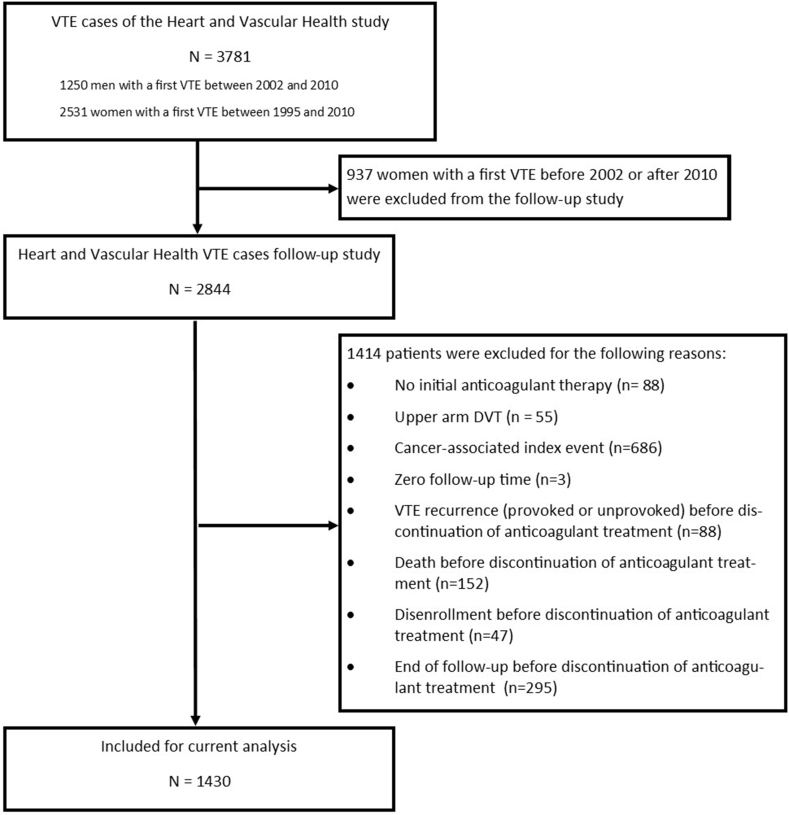
Flow-chart of included patients. DVT, deep venous thrombosis; VTE, venous thromboembolism.

In total, 1430 participants with a first VTE from the HVH study were included in the current analysis. Reasons for exclusion and numbers of excluded participants are shown in Figure 1. The mean age of the participants was 63 years (SD 16 years), and 633 (44.3%) were male. Most participants (49%) had a DVT, 16.3% had a concurrent DVT and PE; 46.7% of the index events were unprovoked. Surgery was the most common provoking factor (54.7% of all provoked cases). The median initial anticoagulant treatment duration was 8.0 months (IQR, 6.4-10.9). Other baseline characteristics are shown in Table 2, and a comparison of baseline characteristics with those from patients of the MEGA study is shown in Supplementary Table 2.

### 3.2 Follow-up and outcomes

The median follow-up duration after discontinuation of anticoagulant therapy was 3.67 years (IQR, 1.38-5.26). During the total follow-up, 187 participants experienced an unprovoked recurrent VTE (including 1 fatal), 67 had a provoked recurrence (including 2 fatal), and 180 participants died. One hundred eleven unprovoked recurrent VTEs, 35 provoked recurrent VTEs, and 117 deaths occurred within the first 2 years of follow-up. Overall, the cumulative incidence of unprovoked recurrent VTE was 5.3% (95% CI, 4.2%-6.6%) at 1 year, 8.5% (95% CI, 7.1%-10%) at 2 years, and 15% (95% CI, 13%-17%) at 5 years after anticoagulant discontinuation. These numbers were 7.2% (95% CI, 5.9%-8.6%), 11% (95% CI, 9.4%-13%), and 19% (95% CI, 17%-21%) for all recurrent VTEs, respectively (Supplementary Table S3).

### 3.3 Predictive performance of model C

**Figure 2:**
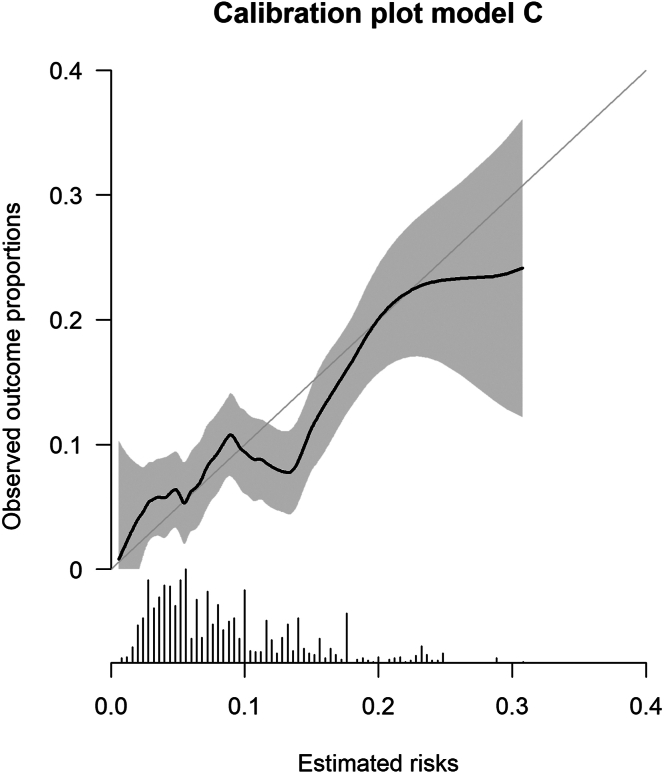
Calibration plot model C. Calibration plot showing estimated risks of recurrent VTE at 2 years according to L-TRRiP model C against observed proportions of recurrent VTE in the HVH study data. The curve including confidence intervals was estimated using pseudo-observations and LOESS smoothing. The gray line indicates perfect calibration. The histogram at the x-axis indicates the distribution of risk estimates. HVH, Heart and Vascular Health; LOESS, locally estimated scatterplot smoothing; VTE, venous thromboembolism.

Model C showed a C-statistic of 0.62 (95% CI, 0.56-0.67). The O/E ratio for model C was 1.00 (95% CI, 0.84-1.17) indicating excellent overall calibration (Table 3). The calibration plot indicated good calibration, with a small underestimation for predicted risks <10% and small overestimation for predicted risks >10% (Figure 2). The AUC showed stable discrimination over the first 7 years; the number of patients followed beyond 7 years was low, resulting in wide CIs (Supplementary Figure S1).

**Table 3:** Predictive performance of L-TRRiP model C.

Analysis	C-index (95% CI)	O/E ratio (95% CI)
Main analysis	0.62 (0.56-0.67)	1.00 (0.84-1.17)
Stratified analysis		
Age <70 y	0.62 (0.55-0.69)	1.11 (0.89-1.32)
Age ≥70 y	0.63 (0.53-0.72)	0.97 (0.71-1.24)
Sensitivity analysis		
Altered surgery definition	0.62 (0.56-0.68)	1.02 (0.85-1.18)
Altered cardiovascular disease history definition	0.62 (0.56-0.67)	1.00 (0.84-1.17)
Without accounting for competing risk of death	0.62 (0.56-0.68)	1.06 (0.89-1.24)
Without censoring of provoked recurrences	0.61 (0.56-0.66)	1.30 (1.11-1.49)
Excluding patients with distal DVT (below vena poplitea)	0.62 (0.55-0.68)	0.95 (0.78-1.12)

CI, confidence interval; DVT, deep venous thrombosis; O/E, observed/expected.

**Figure 3:**
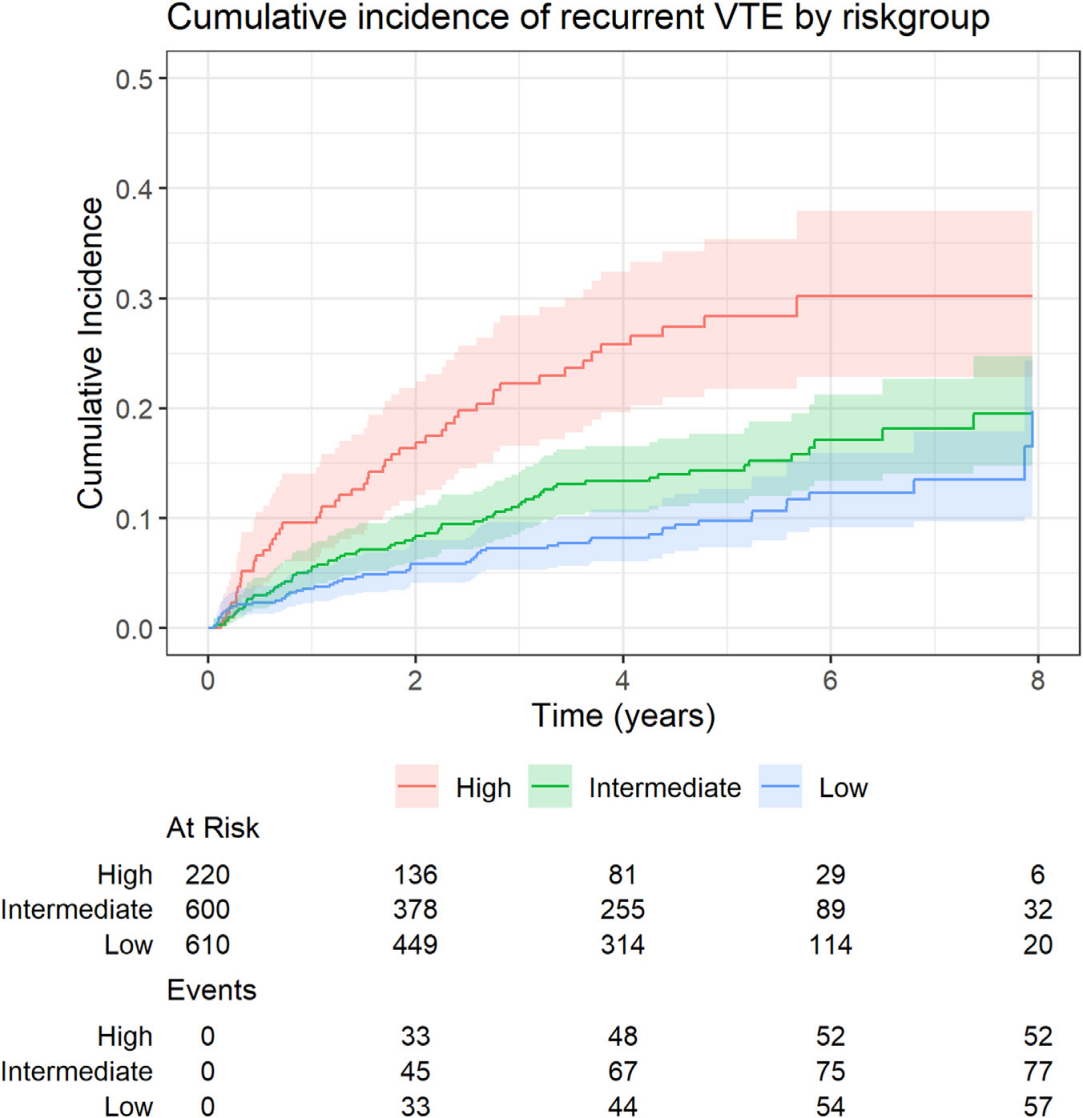
Cumulative incidence of unprovoked recurrent VTE stratified by predicted risk category. Cumulative incidence curves for unprovoked VTE recurrence during follow-up stratified by risk group; a low, intermediate, and high recurrence risk was defined as a predicted recurrence risk according to L-TRRiP model C of <6%, 6% to 14% or >14% within 2 years, respectively. VTE, venous thromboembolism.

**Figure 4:**
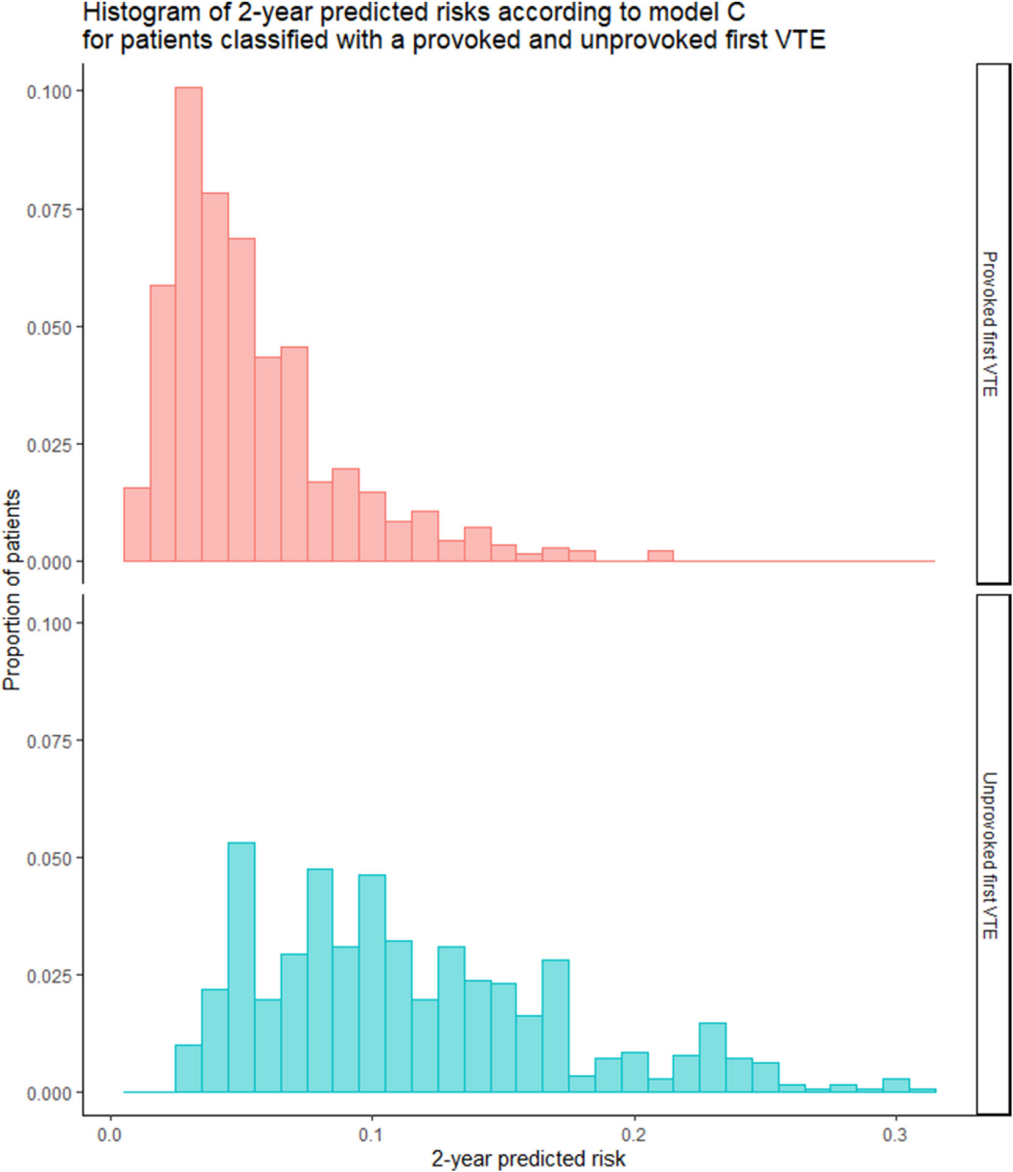
Distribution of predicted risks for patients with a provoked and unprovoked first VTE. Histogram of predicted risks of VTE recurrence at 2 years according to model C for patients with a provoked (top) or unprovoked (bottom) first VTE. VTE, venous thromboembolism.

The cumulative incidence curves for unprovoked recurrent VTE stratified by predicted risk category show that the cumulative incidence of unprovoked recurrent VTE was highest for the high-risk group and lowest for the low-risk group throughout the entire follow-up period (Figure 3, Supplementary Table S3; see Supplementary Figure S2 for the cumulative incidence of the competing risk of death). Figure 4 depicts the distribution of predicted risks for patients with a provoked and unprovoked first VTE and shows there was a significant overlap in the predicted recurrence risks between these groups.

### 3.4 Stratified analysis by age and sensitivity analyses

The model discriminated similarly for participants aged <70 years and ≥70 years and showed good calibration for both (Table 3, Supplementary Figure S3a, c). The sensitivity analysis using an alternate definition of the predictor variables surgery and cardiovascular disease showed similar results as the main analysis (Table 3). There was only a small number of participants for whom the predicted risks were changed due to the altered definitions (Supplementary Table S4). As expected, the analysis ignoring the competing risk of death affected the calibration of the model; the O/E ratios increased due to an increase in the observed incidences of recurrent VTE when ignoring the competing risk of death (Table 3, Supplementary Figure S5). When provoked recurrences were included in the outcome definition, the C-statistic remained similar. However, as expected, the predicted risks seriously underestimated the observed risk of recurrent VTE given that provoked recurrences were not predicted by the models as shown by O/E ratio of 1.30 for model C. The analysis excluding 196 patients with distal DVT (below the level of the vena poplitea) showed results comparable to the main analysis (Table 3). Calibration plots for all sensitivity analysis are provided in Supplementary Figure 4.

### 3.5 Predictive performance and sensitivity analyses of model D

The discriminative ability of model D was slightly lower than that of model C, with a C-statistic of 0.61; 95% CI, 0.55-0.67; the calibration plot and time-dependent AUC of model D were similar (Supplementary Table S5, Supplementary Figures S6, S7). Likewise, the sensitivity analyses for model D showed patterns comparable to model C (Supplementary Table S5, Supplementary Figures S3b, d and S4).

## 4 Discussion

### 4.1 Main findings

This external validation of the L-TRRiP models C and D using the HVH study data showed moderate discrimination and good calibration of both L-TRRiP models. The model performance in the HVH study was lower than that in the original development cohort and slightly lower than that in the previous external validation within the Tromsø study, but higher than in that the external validation within the VTE-PREDICT study, in which a C-statistic of 0.59 was found [8,20]. This is probably due to differences between the populations in baseline characteristics or health care setting.

Although the discriminative ability of the model was moderate, calibration was good, especially in the lower range of predicted risks. The calibration plot suggested a slight underestimation of predicted risks <10% and overestimation for predicted risks >10%, which might indicate overfitting of the model. However, the observed risks did not deviate significantly for predicted risks <10%. Furthermore, only a minority of patients had a predicted risk >10%, resulting in larger uncertainty around the observed outcome incidences as indicated by wider 95% CIs for patients with predicted risks >10%.

For guidance on the decision for long-term anticoagulant treatment, good model calibration is the most important model characteristic. It is essential that the predicted risks around the cutoff value used to determine whether a patient should stop or continue anticoagulant treatment are accurate, as is the case for the L-TRRiP models. Although the exact cutoff value is still under debate, this value likely lies within the lower range of predicted risks (ie, <10% in 2 years); for instance, the European Society of Cardiology (ESC) guideline already categorizes patients based on VTE recurrence risk <3% or >3% per year [21] and previously, the Scientific and Standardization Committee of the International Society of Thrombosis and Haemostasis suggested a recurrence risk >5% within 1 year and 15% within 5 years would not be acceptable to many physicians and patients, and therefore patients are usually discouraged to discontinue anticoagulant therapy because these risks are generally deemed too high [22]. The L-TRRiP models provide accurate risk estimates for these values. However, for patients with a high risk of bleeding combined with a high risk of VTE recurrence, careful balancing between these risks, and therefore accurate prediction of these risks, is essential. The overestimation of predicted risks >10% might limit the application of the L-TRRiP model in these patients.

The analysis stratified by age group showed that discrimination in both age groups was similar, and calibration was good. In addition, we observed that small alterations in the definition of surgery and cardiovascular disease did not impact model performance, indicating the model is robust to such changes. This is an important finding for routine clinical practice where variables will generally not be strictly defined. Furthermore, we observed a significant overlap in the range of predicted risks between patients with a provoked and an unprovoked first event, highlighting that this distinction is not optimal to determine recurrence risk and hence treatment duration, as is current practice and advised in current guidelines [21,[23], [24], [25]].

### 4.2 Strengths and limitations

A strength of the current analysis is that we accounted for the competing risk of death, which we did to prevent overestimation of the cumulative incidence of the outcome. This is crucial since it provides a more reliable assessment of model performance and in particular, model calibration, especially in a setting with a substantial competing risk of death [17,26]. This is also shown by the sensitivity analysis ignoring the competing risk of death, in which the O/E ratio was 1.06, indicating that the observed incidence was indeed overestimated.

A possible limitation of the current validation is that a considerable proportion of the genetic data required for model C was missing in the HVH study. However, based on the reasons for missing genetic data (as described in the Methods section), these data are most likely missing at random and in this case, multiple imputations (as performed) provides valid estimates of model performance [15]. Another limitation is that the HVH study data were collected between 2002 and 2014. Since this period, the treatment for patients with a first VTE has changed. Direct oral anticoagulants (DOACs) replaced vitamin K antagonists (VKAs) as the first choice of treatment, treatment modalities for severe VTE have been improved and guidelines for anticoagulant treatment duration after a first VTE nowadays tend more toward extended anticoagulant treatment in case of a first unprovoked VTE [21,[23], [24], [25]]. We do not expect these differences to affect generalizability of the results of the current study regarding the predictions of recurrent VTEs, as there is no evidence for a difference in VTE recurrence risk between patients initially treated with DOACs or VKAs after anticoagulant discontinuation. However, for extended anticoagulant therapy, DOACs are preferred over VKAs given they are easier to use and have a lower risk of bleeding [4,27]. Since this alters the balance between benefits and harms of extended anticoagulation, model validation in a prospective setting or a management study, in which the benefit of extended anticoagulant treatment based on the estimated recurrence risk is determined, is still needed before this model can be implemented in daily clinical practice. Furthermore, in the current validation, we excluded 342 patients that did not discontinue anticoagulant therapy before disenrollment or end of follow-up. This might have introduced selection bias, especially since a part of these patients with a first VTE were treated with anticoagulation for an extended period (ie, >6 months), although this was not recommended by guidelines at the time of the HVH study. Reasons for extended anticoagulation in these patients are unknown but might include other comorbidities such as atrial fibrillation or VTE-specific factors such as hereditary thrombophilia. Therefore, these patients might differ from the included patients and introduction of some selection bias cannot be excluded. Lastly, the L-TRRiP model can only be used to predict unprovoked recurrences. This was done because unprovoked recurrences are the ones that need to be predicted, as they occur relatively unexpectedly and depend more on inherent characteristics of the patient present at baseline, and extended anticoagulant therapy is the way to prevent these events. In contrast, provoked recurrences depend on future presence of a provoking factor, which cannot be predicted at baseline, and they can be prevented by adequate thromboprophylaxis during exposure to such a provoking factor. Since we only included major provoking factors in our definition of provoked recurrences (ie, malignancy, hormone use, hospitalization, and inpatient surgery or pregnancy), most of these recurrences are preventable by thromboprophylaxis or counseling in these situations. However, thromboprophylaxis cannot prevent all provoked recurrences and therefore, the L-TRRiP model will somewhat underestimate the absolute risk of VTE recurrence. At the same time, excluding the provoked events should increase the predictive performance of the model, as these events are generally unrelated to baseline characteristics. Fortunately, guidelines on thromboprophylaxis have been updated since the time of the HVH study, which should reduce the number of provoked VTE recurrences as compared to our data [28,29].

### 4.3 Implications

The L-TRRiP models can be helpful to guide clinical decision making on duration of anticoagulant therapy after a first VTE. Since model C performs slightly better compared with model D and measurement of the 2 additional predictors (blood group and factor V Leiden) is easy, we would prefer further implementation of model C. Still, the decision to stop or continue treatment related to a certain estimated recurrence risk should be further investigated in a management study in which an individually predicted risk of bleeding should also be taken into account. Currently, the L-TRRiP study is being performed, a clinical trial in which L-TRRiP model C combined with the VTE-BLEED score is used to guide treatment duration in patients with a first VTE (NCT06087952) [30].
