# Supplementary material for: External validation of the Leiden Thrombosis Recurrence Risk Prediction models (L-TRRiP) for the prediction of recurrence after a first venous thrombosis in the Heart and Vascular Health study
Source: Res Pract Thromb Haemost. 2024 Oct 29;8(8):102610. doi: 10.1016/j.rpth.2024.102610 (PMC11617231; doi:10.1016/j.rpth.2024.102610)
Supplement: Supplemental Data [file mmc1.docx]

Supplementary material

- **Supplementary table 1 Predictor variable definition**
- **Supplementary table 2 Baseline characteristics within the MEGA and HVH studies**
- **Supplementary table 3 Cumulative incidence of VTE recurrence at 1, 2, 5 and 10 years for unprovoked and all recurrent VTE, stratified by risk category**
- **Supplementary table 4 Number of participants for predictors surgery and cardiovascular disease according to main- and sensitivity analysis**
- **Supplementary figure 1 Time dependent AUC model C**
- **Supplementary figure 2 Cumulative incidence of the competing risk of death stratified by predicted VTE recurrence risk category**
- **Supplementary figure 3 Calibration plots for model C and D for participants <70 and ≥ 70 years old**
- **Supplementary figure 4 Calibration plots for model C and D for the sensitivity analyses**
- **Supplementary figure 5 Difference between Kaplan-Meier and Aalen-Johansen estimates of the observed outcome probabilities**
- **Supplementary figure 6 Calibration plot model D**
- **Supplementary figure 7 Time dependent AUC model D**
- **R-code of main analysis**

**Tables**

| **Supplementary table 1** Definition of predictor variables in the MEGA study and main analysis of current study and sensitivity analysis | | | |
| --- | --- | --- | --- |
| **Variable** | **Definition MEGA study (derivation cohort of the L-TRRiP models)** | **Definition main analysis** | **Altered definition sensitivity analysis** |
| Sex | Male or female | As in MEGA study | n.a. |
| Type of 1^st^ VTE  (DVT, PE, PE + DVT) | Only DVT, only PE or DVT and PE; based on revision of medical records and radiology reports of diagnosis | As in MEGA study | n.a. |
| Location of DVT  (Proximal vs popliteal DVT) | Proximal (above v. poplitea (or distal (v. poplitea or below), based on radiology report of diagnosis | As in MEGA study | n.a. |
| Surgery | Surgery within 3 months before VTE, self-reported via questionnaire | Inpatient or outpatient surgical procedures within 3 months before VTE, identified through ICD codes | As main analysis + major diagnostic procedures (e.g., coronary catheterization) within 3 months before VTE |
| Pregnancy/puerperium | Pregnancy at time of VTE or end of pregnancy within 3 months before VTE, self-reported via questionnaire | As in MEGA study, identified through ICD codes | n.a. |
| Hormone use | Hormone replacement therapy or hormonal contraceptive use (all types) at time of VTE, self-reported via questionnaire | As in MEGA study, identified through ICD codes | n.a. |
| Plaster cast | Any plaster cast within 3 months before VTE, self-reported via questionnaire | As in MEGA study, identified through ICD codes | n.a. |
| Immobility in bed, in hospital | Confinement to bed >=3 days in hospital, within 3 months before VTE, self-reported via questionnaire | Any hospitalization >= 3 days within 3 months before VTE, identified through ICD codes | n.a. |
| Cardiovascular disease | History of heart failure angina pectoris, arterial insufficiency of the legs or acute myocardial infarction, self-reported via questionnaire | Myocardial infarction, angina pectoris, heart failure, percutaneous transluminal coronary angioplasty (PTCA), coronary artery bypass graft (CABG), arterial insufficiency of the legs or carotid endarterectomy before VTE, identified through ICD codes | Myocardial infarction, angina pectoris, heart failure or arterial insufficiency of the legs |
| Blood group, non-O vs O | Blood group determined via SNPs (rs8176719, rs7853989, rs8176749, rs8176750) | As in MEGA study | n.a. |
| Factor V Leiden mutation | Homozygous or heterozygous (vs 1691GG) | As in MEGA study | n.a. |

| **Supplementary table 2** Comparison of baseline characteristics with characteristics from MEGA study | | |
| --- | --- | --- |
|  | **HVH-study** | **MEGA study (development)** |
|  | *n (%) or mean (SD)* | |
| Total number of participants | 1430 | 3,750 |
| Age, years (SD) | 63.2 (15.9) | 48 (13) |
| Male sex | 633 (44.3) | 1,684 (45) |
| Type of first event |  |  |
| DVT only | 701 (49.0) | 2,231 (59) |
| PE only | 496 (34.7) | 1,184 (32) |
| PE + DVT | 233 (16.3) | 335 (9) |
| Location of first DVT (if applicable) |  |  |
| Proximal DVT | 436 (32.6) | 634 (22.1) |
| Popliteal or distal DVT^1^ | 406 (30.3) | 1048 (36.6) |
| Provoked first event^2,3^ | 720 (50.3) | 2,592 (69) |
| Trauma | 179 (12.6) | N.S. |
| Surgery | 394 (27.7) | N.S. |
| Hospitalization > 3 days | 233 (16.3) | N.S. |
| Trauma, surgery, immobilization | N.S. | 1,458 (39) |
| Plaster cast | 86 (6.1) | 198 (5) |
| Pregnancy, puerperium | 15 (1.0) | 160 (4) |
| Hormone use | 184 (12.9) | 1,181 (31) |
| Prolonged travel | N.S. | 681 (18) |
| Unprovoked first event | 710 (49.7) | 1,082 (29) |
| History of cardiovascular disease | 275 (19.2) | 194 (5.5) |
| Non-O blood group | 626 (67.0) | 2,464 (72.0) |
| Factor V Leiden mutation | 110 (11.8) | 568 (16.5) |
| Abbreviations: N.S.: not specified.  Continues variables are denoted as mean (SD), categorical variables are denoted as number (%)  ^1^ Indicates DVT at the level of the vena poplitea or below.  ^2^ Index VTE was classified as provoked in case of major trauma, surgery, plaster cast, pregnancy, hospitalization > 3 days or hormone use in the 3 months prior to the index event in the HVH study; trauma, surgery, immobilization at home or in hospital, plaster cast, pregnancy, hormone use or prolonged travel in the MEGA study; and trauma, surgery, immobilization at home or in hospital, pregnancy and hormone use in the Tromsø study.  ^3^ Total of all risk factors below exceeds the amount of provoking first events since multiple provoking risk factors could be present concomitantly. | | |

| **Supplementary table 3** Cumulative incidence and 95% CI of recurrent VTE overall and per risk group after one, two, five and ten years of follow-up in the HVH-study | | | | |
| --- | --- | --- | --- | --- |
|  | **At 1 year** | **2 years** | **5 years** | **10 years** |
| *Unprovoked recurrent VTE* | | | | |
| Overall | 5.3% (4.2-6.6%) | 8.5% (7.1-10%) | 15% (13-17%) | 24% (17-31%) |
| Low risk | 3.6% (2.3-5.3%) | 5.8% (4.1-8.0%) | 9.8% (7.3-13%) | 20% (11-30%) |
| Intermediate risk | 5.6% (3.9-7.7%) | 8.4% (6.2-11%) | 14% (11-18%) | 19% (15-25%) |
| High risk | 9.6% (6.1-14%) | 16% (12-22%) | 28% (22-35%) | 87% (0-100%) |
| *All recurrent VTE* | | | | |
| Overall | 7.2% (5.9-8.6%) | 11% (9.4-13%) | 19% (17-21%) | 32% (25-39%) |
| Low risk | 4.7% (3.2-6.6%) | 7.2% (5.3-9.6%) | 13% (10-16%) | 25% (16-35%) |
| Intermediate risk | 8.6% (6.4-11%) | 12% (9.5-15%) | 20% (16-23%) | 31%(23-39%) |
| High risk | 10% (6.8-15%) | 19% (14-24%) | 32% (26-39%) | 87% (0-100%) |

| **Supplementary table 4** number of participants with prediction factor surgery and cardiovascular disease according to definition from main analysis and sensitivity analysis | | |
| --- | --- | --- |
| **Definition according to** | **Main analysis** | **sensitivity analysis** |
|  | *n (%)* | *n (%)* |
| Surgery | 394 (27.7) | 423 (29.7) |
| History of cardiovascular disease | 275 (19.2) | 261 (18.3) |

| **Supplementary table 5** Predictive performance of L-TRRiP model D | | |
| --- | --- | --- |
|  | **C-index (95%CI)** | **O/E ratio (95%CI)** |
| Main analysis | 0.61 (0.55-0.67) | 1.09 (0.91-1.27) |
| *Stratified analysis* | | |
| Age <70 years | 0.60 (0.52-0.68) | 1.17 (0.95-1.40) |
| Age >= 70 years | 0.63 (0.53-0.73) | 0.94 (0.69-1.20) |
| *Sensitivity analysis* | | |
| Altered surgery definition | 0.61 (0.56-0.67) | 1.10 (0.92-1.30) |
| Altered cardiovascular disease history definition | 0.61 (0.55-0.67) | 1.09 (0.91-1.26) |
| Without accounting for competing risk of death | 0.61 (0.55-0.67) | 1.15 (0.96-1.34) |
| Without censoring of provoked recurrences | 0.60 (0.55-0.65) | 1.40 (1.21-1.61) |
| Excluding patients with distal DVT (below v. poplitea) | 0.61 (0.55-0.67) | 1.04 (0.85-1.22) |

**Figures**


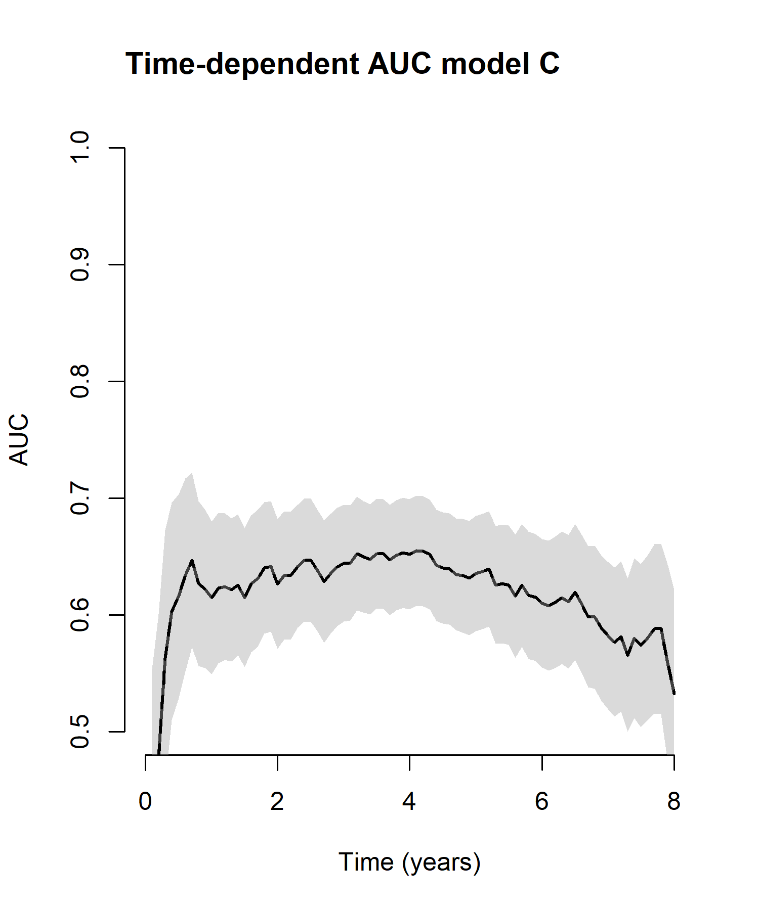


**Supplementary figure 1** Time dependent AUC model C.
Plot of the time-dependent AUC of model C over the follow-up time. The black line indicates the estimated AUC at a certain point of follow-up time, the grey area represents the 95% confidence interval.


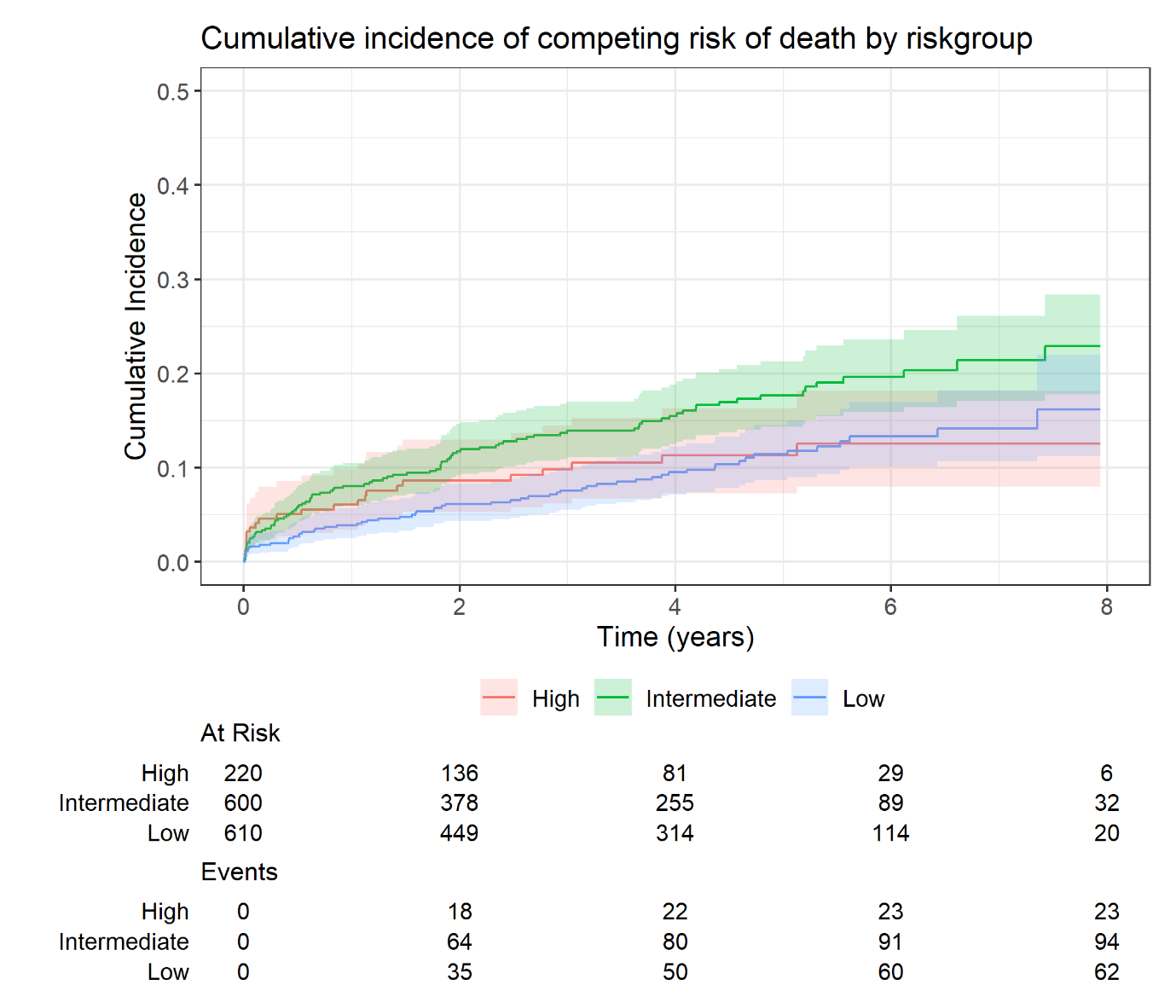


**Supplementary figure 2** Cumulative incidence curves for the competing risk of death (death before VTE recurrence) during follow-up stratified by risk group; a low, intermediate, and high recurrence risk was defined as a predicted recurrence risk according to L-TRRiP model C of <6%, 6-14% or >14% within two years, respectively.

|  | **Model C** | **Model D** |
| --- | --- | --- |
| **Age < 70 years** | a.  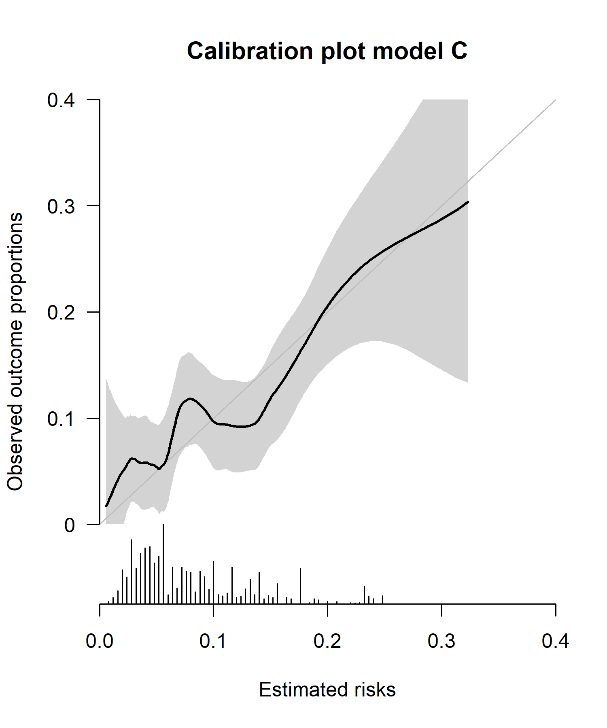 | b.  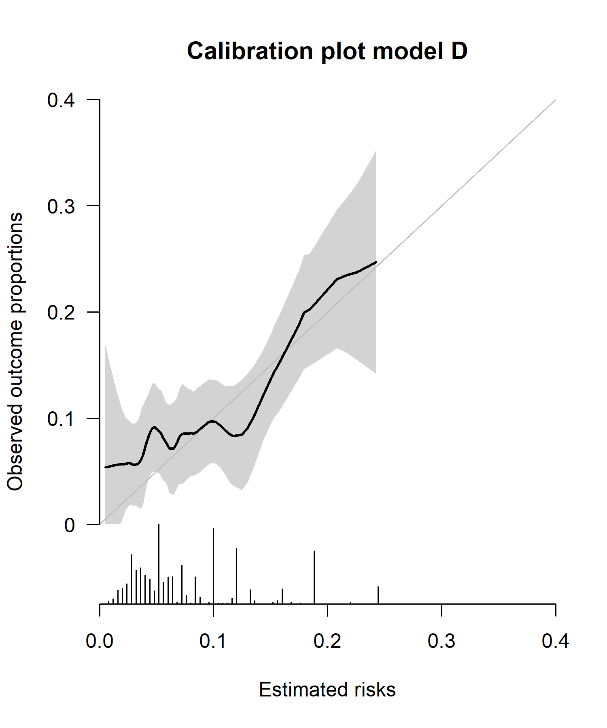 |
| **Age ≥ 70 years** | c.  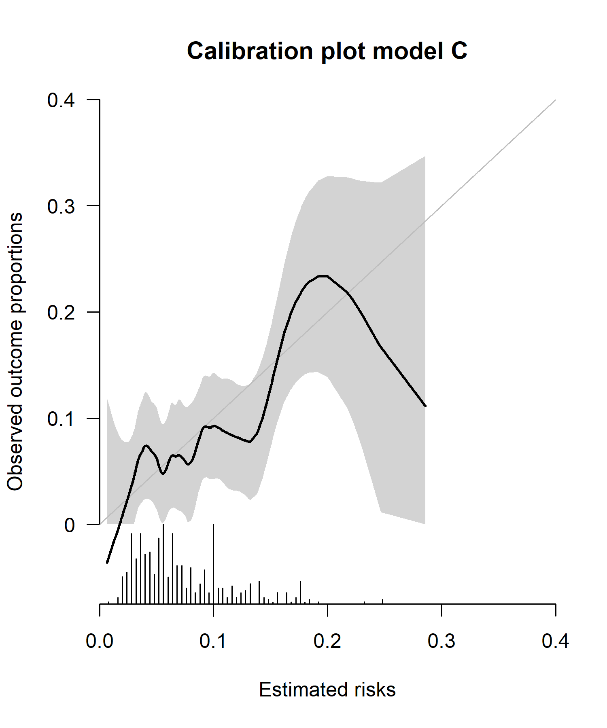 | d.  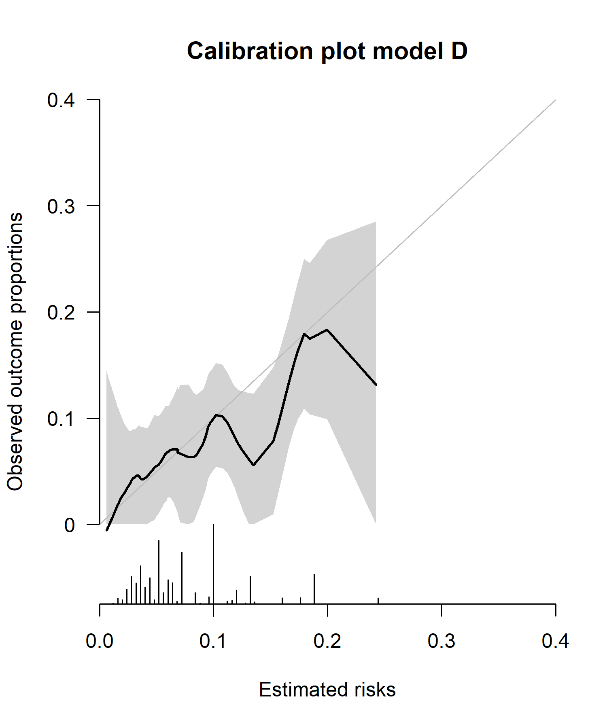 |

**Supplementary figure 3** **a-d** Calibration plots for model C and D stratified by age.
Calibration plots showing estimated risks of recurrent VTE at two years according to L-TRRiP model C and D against observed proportions of recurrent VTE in the HVH study data stratified by age <70 or ≥ 70 years. The curves including confidence intervals were estimated using pseudo-observations and LOESS smoothing. The grey lines indicate perfect calibration. The histograms at the x-axis indicate the distribution of risk estimates.

|  | **Model C** | **Model D** |
| --- | --- | --- |
| **Altered surgery definition** | 1. 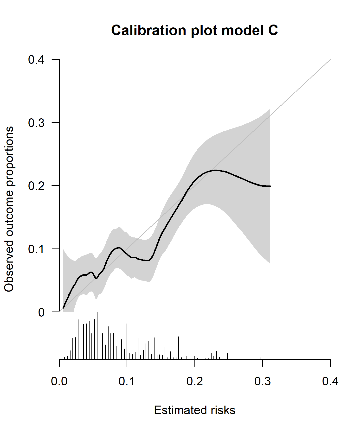 | 1. 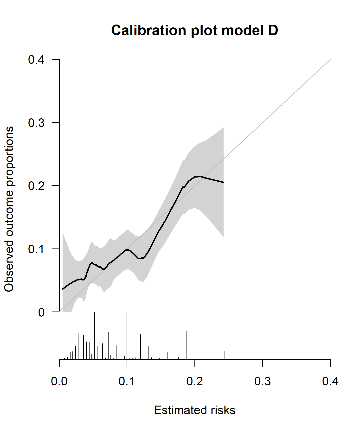 |
| **Altered cardiovascular disease history definition** | 1. 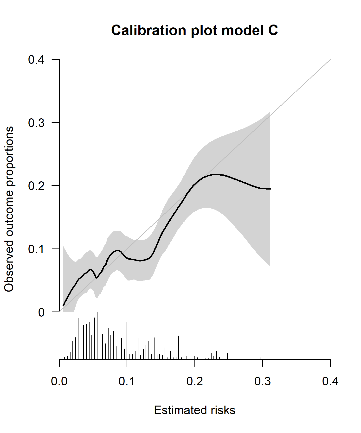 | 1. 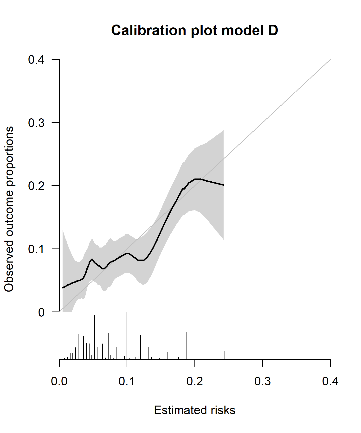 |
| **Without censoring of provoked recurrences** | 1. 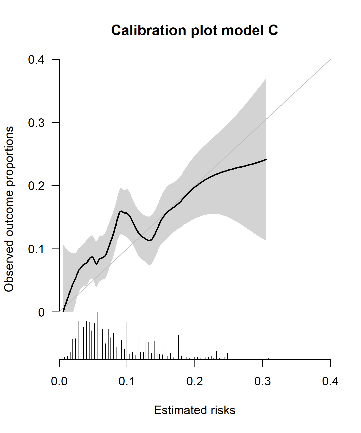 | 1. 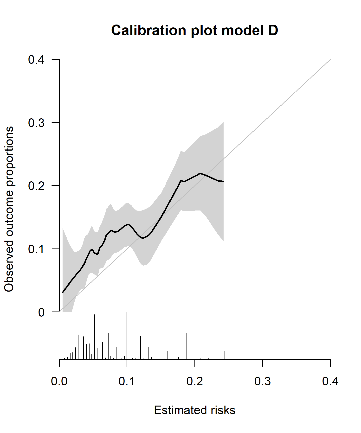 |
| **Excluding patients with distal DVT (below v. poplitea)** | 1. 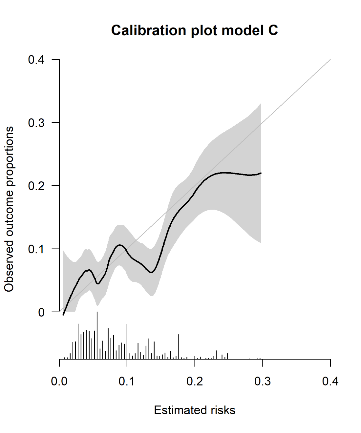 | 1. 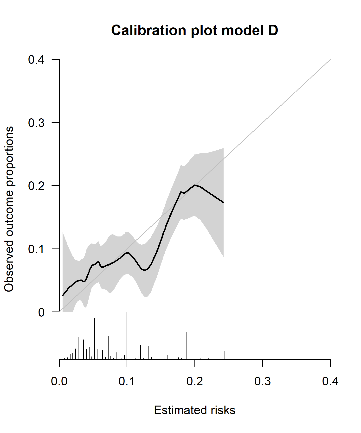 |

**Supplementary figure 4** **a-h** Calibration plots for model C and D for each sensitivity analysis.
Calibration plots showing estimated risks of recurrent VTE at two years according to L-TRRiP model C and D against observed proportions of recurrent VTE in the HVH study data for each of the performed sensitivity analysis. The curves including confidence intervals were estimated using pseudo-observations and LOESS smoothing. The grey lines indicate perfect calibration. The histograms at the x-axis indicate the distribution of risk estimates.


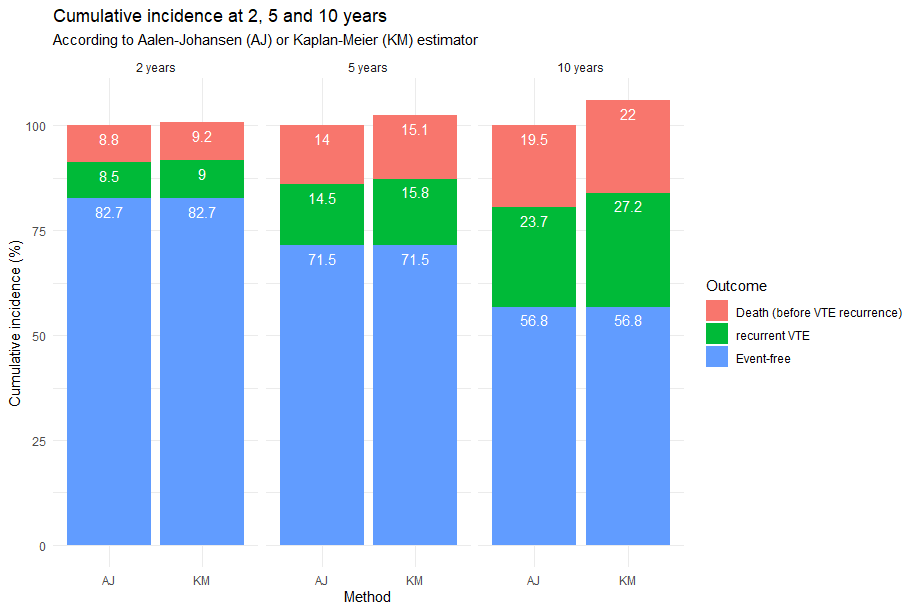


**Supplementary figure 5** Difference between Kaplan-Meier and Aalen-Johansen estimates of the observed outcome probabilities.
 Bar plot indicating the difference in the estimated cumulative incidence of recurrent VTE (green), death (red) and event-free survival (blue) between analysis accounting for the competing risk of death using the Aalen Johansen estimator and without accounting for the competing risk of death using the Kaplan Meier estimator at 2, 5 and 10 years of follow-up. When using the Kaplan-Meier estimator (ignoring the competing risk of death by censoring deceased participants) the cumulative incidence of recurrent VTE is overestimated especially after a longer follow-up period, which in turn would affect model calibration as the observed incidences are not estimated correctly when ignoring the competing risk of death.


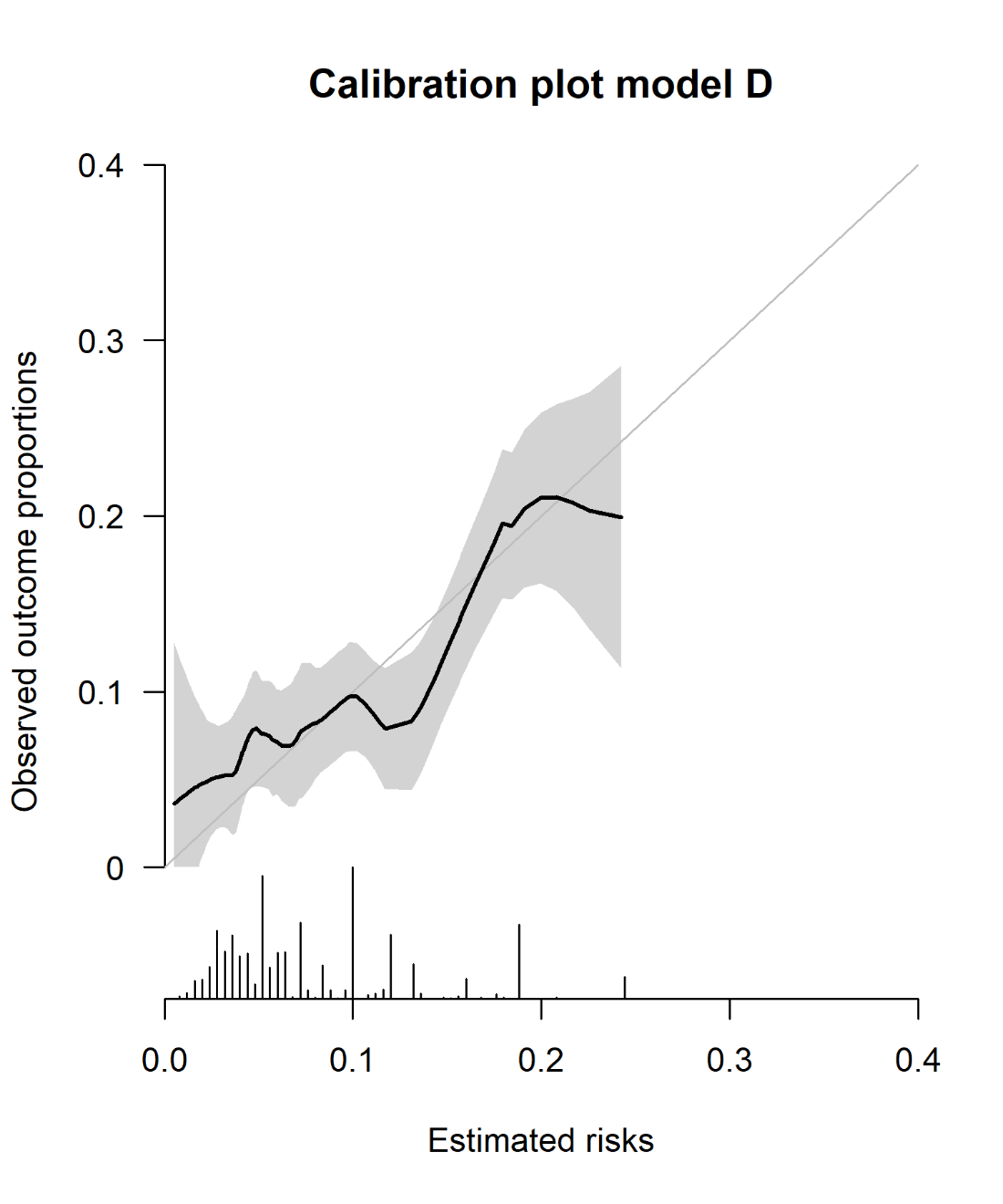


**Supplementary figure 6** Calibration plot model D.
Calibration plot showing estimated risks of recurrent VTE at two years according to L-TRRiP model D against observed proportions of recurrent VTE in the HVH study data. The curve including confidence intervals was estimated using pseudo-observations and LOESS smoothing. The grey line indicates perfect calibration. The histogram at the x-axis indicates the distribution of risk estimates.


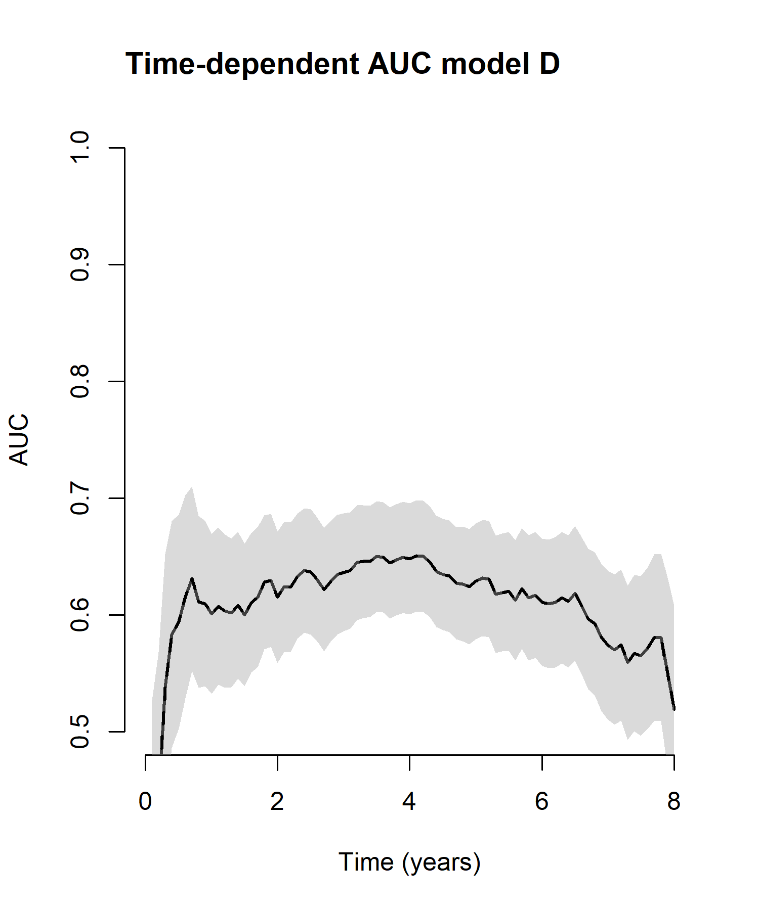


**Supplementary figure 7** Time dependent AUC model D.
Plot of the time-dependent AUC of model D over the follow-up time. The black line indicates the estimated AUC at a certain point of follow-up time, the grey area represents the 95% confidence interval.

R code external validation L-TRRiP - cleaned version for supplement

Louise Burggraaf and Kerri Wiggins

2023-12-14

This document contains the code for the main-analysis and the analysis without competing risks. For the analysis stratified by age and the sensitivity analysis an altered data set was used.

## Packages

#install and load packages
#install.packages ("pacman")
library(pacman)
p_load(cmprsk, dynpred, ggsurvfit, haven, mice, lattice, pec, riskRegression, rsample, survival, tableone, tidyverse)

## Data dictonairy

| Variable name | Description | variable levels |
| --- | --- | --- |
| male | Indicator whether case is male | 0 = no, 1 = yes |
| lft | Age in years | n.a. |
| oper3mnd | Surgery in past 3 months | 0 = no, 1 = yes |
| gips3mnd | Cast in past 3 months | 0 = no, 1 = yes |
| bedzk3mnd | Hospitalization >= 3 days in past 3 months | 0 = no, 1 = yes |
| zwan3mnd | Pregnancy in past 3 months | 0 = no, 1 = yes |
| hormoon | Hormone use at time of index VTE | 0 = no, 1 = yes |
| discardio | History of cardiovascular disease | 0 = no, 1 = yes |
| fvl | Factor V Leiden mutation (homo or heterozygous) | 0 = no, 1 = yes |
| blgroup | Bloedgroup non O (i.e. A, B or AB) | 0 = no, 1 = yes |
| TypeVT | Type of index VTE | 1 = DVT, 2 = PE, 3 = PE + DVT |
| DVT_loc | Location of DVT | 0 = PE only, 1 = proximal, 2 = distal (popliteal or below) |
| unprovoked | Indicator whether index event was unprovoked | 0 = no, 1 = yes |
| trau3mo | Trauma in past 3 months | 0 = no, 1 = yes |
| fuduur_J | follow-up in days (from stop of ac use) | n.a. |
| status | Status at end of follow-up | 0 = censor, 1 = rVTE, 2 = Death |
| status_unprov | Status at end of follow-up censoring provoked recurrence | 0 = censor, 1 = rVTE (unprovoked only), 2 = death |

## Initial data analysis and data transformations

head(df)

# Check for missing values
md.pattern(df)

# Set categorical variables to be treated as factors
df$male <- as.factor(df$male)
df$TypeVT <- as.factor(df$TypeVT)
df$oper3mnd <- as.factor(df$oper3mnd)
df$bedzk3mnd <- as.factor(df$bedzk3mnd)
df$gips3mnd <- as.factor(df$gips3mnd)
df$zwan3mnd <- as.factor(df$zwan3mnd)
df$hormoon <- as.factor(df$hormoon)
df$unprovoked <- as.factor(df$unprovoked)
df$discardio <- as.factor(df$discardio)
df$fvl <- as.factor(df$fvl)
df$blgroup <- as.factor(df$blgroup)
df$DVT_loc <- as.factor(df$DVT_loc)

##Transform status & follow-up; censor provoking events; compute status & fu at 2 years
#Compute fu-time in years
df$time <- df$fuduur_J/365.25
df<-df[, !names(df) %in% c("fuduur_J")]

#Create time and status variable at two years
df$time_2yr <- ifelse (df$time<2, df$time, 2)
df$status_unprov_2yr <- ifelse (df$time<2, df$status_unprov, 0)

#transform status unprovoked with censoring of death (for analysis without competing event)
df$status_unprov_dcens <- ifelse (df$status_unprov ==1, df$status_unprov, 0)

## Creating imputed datasets

#Compute cumulative hazard to use for imputation
df$ch<-nelsonaalen (df, time, status_unprov_dcens)

#Preparation
set.seed(1234) #set seed to make the MI proces reproducable after replication.
imp <- mice(df,
 m=10, #create 10 imputed sets
 maxit=0, print=F)#zero iterations, to enable altering of mi methods and run it afterwards

predM<-imp$predictorMatrix
meth<-imp$method
predM
meth #logreg for 2 factor variables, polyreg for DVT-Loc, this is correct

#Set values of the different time/status values to 0 in predictor matrix (only use cumulative hazard for the outcome)
predM[,c("status", "time", "time_2yr", "status_unprov", "status_unprov_2yr", "status_unprov_dcens" )] <-0
head(predM) #these variables are now indeed set to zero

#Create 10 imputed datasets with 40 iterations using the defined predictor matrix
df_imp <- mice(df, m=10, maxit=40, predictorMatrix=predM, method = meth, print=F)

plot(df_imp) #check plots to see whether convergence is reached at the later iterations.

#transform to one df with long format for further calculations
df_long <- mice::complete(df_imp, action = "long", include= T)

rm(imp, predM, meth, df_imp)

## Compute predicted risk scores in imputed dataset

#compute variable for PE_only, PE+DVT and distalDVT
df_long$PE_only <- ifelse (df_long$TypeVT==2,1,0)
df_long$PE_DVT <- ifelse (df_long$TypeVT==3,1,0)
df_long$distal_dvt <- ifelse(df_long$DVT_loc==2,1,0)

#compute prognostic score according to model c and d;
#coefficients from timp et al. correction. Plos Med 2021.
#note that due to setting variables to factors a transformation to numeric values is required for calculation since the as.numeric function returns 1 and 2 instead of 0 and 1 -1 is added to the calculation.
df_long$xb_modc <- (0.63*(as.numeric(df_long$male)-1)-0.61*df_long$PE_only +0.32*df_long$PE_DVT -0.46*df_long$distal_dvt-0.51*(as.numeric(df_long$oper3mnd)-1) -1.49*(as.numeric(df_long$zwan3mnd)-1) -0.67*(as.numeric(df_long$hormoon)-1)-0.79*(as.numeric(df_long$gips3mnd)-1) -0.31*(as.numeric(df_long$bedzk3mnd)-1) -0.36*(as.numeric(df_long$discardio)-1) +0.24*(as.numeric(df_long$blgroup)-1)
+0.40*(as.numeric(df_long$fvl)-1))
df_long$xb_modd <- (0.68*(as.numeric(df_long$male)-1) -0.69*df_long$PE_only +0.31*df_long$PE_DVT -0.49*df_long$distal_dvt -0.52*(as.numeric(df_long$oper3mnd)-1) -1.44*(as.numeric(df_long$zwan3mnd)-1) -0.62*(as.numeric(df_long$hormoon)-1) -0.83*(as.numeric(df_long$gips3mnd)-1) -0.34*(as.numeric(df_long$bedzk3mnd)-1) -0.37*(as.numeric(df_long$discardio)-1))

#compute 2 years predicted recurrence risks according to model c and d
#baseline recurrence free probability from timp et al. correction. PlOs Med 2021.
df_long$pred_2jr_modc <- 1-0.9235595^exp(df_long$xb_modc)
df_long$pred_2jr_modd <- 1-0.9019939^exp(df_long$xb_modd)

#Extract mean predicted 2 years risks per patient to use for calibration
mean_pred_2jr_modc<- df_long %>% group_by(.id) %>%
 summarise(mean_pred_2jr_modc = mean(pred_2jr_modc, na.rm=T))
mean_pred_2jr_modd<-df_long %>% group_by(.id) %>%
 summarise(mean_pred_2jr_modd = mean(pred_2jr_modd, na.rm=T))
#add mean predicted risks to non imputed dataframe
df<-cbind(df, mean_pred_2jr_modc,mean_pred_2jr_modd) #add mean predicted risks to df
rm(mean_pred_2jr_modc,mean_pred_2jr_modd)

##Convert long dataset back to mids-type for further analysis
df_long_mids<-as.mids(df_long)

## Table 2: Baseline characteristics of study population

Note: table 1 is computed on the original (non-imputed) data

#Variables to summarize
vars_t1 <-c("lft", "male", "TypeVT", "DVT_loc", "unprovoked", "trau3mo", "oper3mnd", "bedzk3mnd", "gips3mnd", "zwan3mnd", "hormoon", "discardio", "blgroup", "fvl")

#Categorical variables
catVars_t1 <-c("male", "TypeVT", "DVT_loc", "unprovoked", "trau3mo", "oper3mnd", "bedzk3mnd", "gips3mnd", "zwan3mnd", "hormoon", "discardio", "blgroup", "fvl")

#Create table 2
tab1 <- CreateTableOne(vars = vars_t1, data=df, factorVar=catVars_t1)
summary(tab1) #includes percentage of missing values

rm(vars_t1, catVars_t1)

## Table 3: Predictive performance of L-TRRiP model C and D 4 predictive performance

Code for predictive performance, calibration plots and AUCt plots was adapted from the github provided by *van Geloven et al. Validation of prediction models in the presence of competing risks: a guide through modern methods. BMJ 2022* (PMID: 35609902)

Link to github: [link] <https://github.com/survival-lumc/ValidationCompRisks>

### Main analysis

#Preparations
horizon <- 2 #prediction horizon of 2 years
primary_event <- 1 #primary_event = rVTE which is coded as 1
results <-data.frame()#create empty data frame to store the results
m <-10 #number of imputations

#Compute c-index and O/E ratio for each imputed dataset
for(i in 1:m){
 dat.temp<-complete(df_long_mids,i)

 ##C-index
 #model C
 fit_csh_c <- CSC(formula = (Hist(time, status_unprov)~xb_modc),
 data = dat.temp,
 cause = primary_event)
 cindex_csh_c<-pec::cindex(
 object=fit_csh_c,
 formula = Hist(time,status_unprov)~1,
 cause = primary_event,
 eval.times=horizon,
 data=dat.temp,
 )$AppCindex$CauseSpecificCox

 #bootstrap for standard error of cindex
 B<-100 #(nr of bootstraps)
 set.seed(1234)
 boots_ls_c <- lapply(seq_len(B), function(b) {
 # Resample validation data
 dat.temp_boot <- dat.temp[sample(nrow(dat.temp), replace = TRUE), ]
 # Get cindex on boot validation data
 cindex_c_boot <- pec::cindex(
 object = fit_csh_c,
 formula = Hist(time, status_unprov) ~ 1,
 cause = primary_event,
 eval.times = horizon,
 data = dat.temp_boot,
 verbose = FALSE
 )$AppCindex$CauseSpecificCox
 cbind.data.frame("cindex"=cindex_c_boot)
 })
 dat.temp_boots_c <-do.call(rbind.data.frame,boots_ls_c)
 #extract standard error
 cindex_c_se <- sd(dat.temp_boots_c$cindex) #se is SD of the 100 bootstraps

 #model D
 fit_csh_d <- CSC(formula = (Hist(time, status_unprov)~xb_modd),
 data = dat.temp,
 cause = primary_event)

 cindex_csh_d<-pec::cindex(
 object=fit_csh_d,
 formula = Hist(time,status_unprov)~1,
 cause = primary_event,
 eval.times=horizon,
 data=dat.temp,
 )$AppCindex$CauseSpecificCox

#bootstrap for standard error of cindex
 B<-100 #(nr of bootstraps)
 set.seed(1234)
 boots_ls_d <- lapply(seq_len(B), function(b) {
 # Resample validation data
 dat.temp_boot <- dat.temp[sample(nrow(dat.temp), replace = TRUE), ]
 # Get cindex on boot validation data
 cindex_d_boot <- pec::cindex(
 object = fit_csh_d,
 formula = Hist(time, status_unprov) ~ 1,
 cause = 1,
 eval.times = horizon,
 data = dat.temp_boot,
 verbose = FALSE
 )$AppCindex$CauseSpecificCox
 cbind.data.frame("cindex"=cindex_d_boot)
 })
 dat.temp_boots_d <-do.call(rbind.data.frame,boots_ls_d)
 #extract standard error
 cindex_d_se <- sd(dat.temp_boots_d$cindex)

 ##Observed/expected ratio
 #Calculate Aalen-Johansen estimate as 'observed'
 obj<-summary(survfit.formula(Surv(time,as.factor(status_unprov))~1, data=dat.temp), times=horizon)
 aj<-list(
 "obs"=obj$pstate[, primary_event + 1],
 "se" =obj$std.err[, primary_event + 1]
 )
 #Calculate OE model C & D
 OE_C <- aj$obs/mean(dat.temp$pred_2jr_modc)
 OE_D <- aj$obs/mean(dat.temp$pred_2jr_modd)

 # For the confidence interval we use the method proposed in Debray et al. (2017) doi:10.1136/bmj.i6460
 # The se is obtained from the 95% CI
 alpha <- 0.05
 OE_C_ll95CI <- exp(log(OE_C) - qnorm(1 - alpha / 2) * aj$se / aj$obs) #lower limit of 95% CI O/E model C
 OE_D_ll95CI <- exp(log(OE_D) - qnorm(1 - alpha / 2) * aj$se / aj$obs) #lower limit of 95% CI O/E model D
 #Compute se
 OE_C_se <- (OE_C-OE_C_ll95CI)/1.96
 OE_D_se <- (OE_D-OE_D_ll95CI)/1.96

 #add results to dataframe
 result_row<- data.frame(Dataset = i,
 CIndex_C = cindex_csh_c, CIndex_C_se = cindex_c_se,
 CIndex_D = cindex_csh_d, CIndex_D_se = cindex_d_se,
 OE_C = OE_C, OE_C_se = OE_C_se,
 OE_D = OE_D, OE_D_se = OE_D_se)
 results<- rbind(results, result_row)
}

rm(aj, boots_ls_c, boots_ls_d, dat.temp, dat.temp_boots_c, dat.temp_boots_d, fit_csh_c, fit_csh_d, obj, result_row, alpha, B, cindex_csh_c, cindex_c_se, cindex_csh_d, cindex_d_se, i, OE_C, OE_C_se, OE_C_ll95CI, OE_D, OE_D_se, OE_D_ll95CI)

#### Pool results according to rubin’s rules

see: [link] <https://bookdown.org/mwheymans/bookmi/rubins-rules.html>

#Cindex_C
cindex_c_varb <- var(results$CIndex_C)
cindex_c_varw <- mean(results$CIndex_C_se^2)
cindex_c_vartot <- cindex_c_varw + cindex_c_varb + (cindex_c_varb/m)
cindex_c_se_pooled <- sqrt(cindex_c_vartot)
cindex_c_pooled_summary <- cbind(
 "Cindex" = mean(results$CIndex_C),
 "lower .95" = mean(results$CIndex_C)- 1.96*cindex_c_se_pooled,
 "upper .95" = mean(results$CIndex_C)+ 1.96*cindex_c_se_pooled
 )
cindex_c_pooled_summary #Pooled C-statistic model C
rm(cindex_c_varb, cindex_c_varw, cindex_c_vartot, cindex_c_se_pooled)

#Cindex_D
cindex_d_varb <- var(results$CIndex_D)
cindex_d_varw <- mean(results$CIndex_D_se^2)
cindex_d_vartot <- cindex_d_varw + cindex_d_varb + (cindex_d_varb/m)
cindex_d_se_pooled <- sqrt(cindex_d_vartot)
cindex_d_pooled_summary <- cbind(
 "Cindex" = mean(results$CIndex_D),
 "lower .95" = mean(results$CIndex_D)- 1.96*cindex_d_se_pooled,
 "upper .95" = mean(results$CIndex_D)+ 1.96*cindex_d_se_pooled
)
cindex_d_pooled_summary #Pooled C-statistic model D
rm(cindex_d_varb, cindex_d_varw, cindex_d_vartot, cindex_d_se_pooled)

#OE_C
OE_C_varb <- var(results$OE_C)
OE_C_varw <- mean(results$OE_C_se^2)
OE_C_vartot <- OE_C_varw + OE_C_varb + (OE_C_varb/m)
OE_C_se_pooled <- sqrt(OE_C_vartot)
OE_C_pooled_summary <- cbind(
 "OE" = mean(results$OE_C),
 "lower .95" = mean(results$OE_C)- 1.96*OE_C_se_pooled,
 "upper .95" = mean(results$OE_C)+ 1.96*OE_C_se_pooled
)
OE_C_pooled_summary #Pooled O/E ratio model C
rm(OE_C_varb, OE_C_varw, OE_C_vartot, OE_C_se_pooled)

#OE_D
OE_D_varb <- var(results$OE_D)
OE_D_varw <- mean(results$OE_D_se^2)
OE_D_vartot <- OE_D_varw + OE_D_varb + (OE_D_varb/m)
OE_D_se_pooled <- sqrt(OE_D_vartot)
OE_D_pooled_summary <- cbind(
 "OE" = mean(results$OE_D),
 "lower .95" = mean(results$OE_D)- 1.96*OE_D_se_pooled,
 "upper .95" = mean(results$OE_D)+ 1.96*OE_D_se_pooled
)
OE_D_pooled_summary #Pooled O/E ratio model D
rm(OE_D_varb, OE_D_varw, OE_D_vartot, OE_D_se_pooled)

### Sensitivity analysis without competing risks

#Preparations
results_s1 <-data.frame()#create empty data frame to store the results_s1 of the analysis within the imputated data

#compute c-index and O/E ratio for each imputed dataset
for(i in 1:m){
 dat.temp<-complete(df_long_mids,i)

 #create status indicator at two years were death is censored;
 #since horizon for main analysis is two years, we'll use data for 2 years for this analysis as well.
 dat.temp$status_unprov_2yr_dcens <- ifelse (dat.temp$status_unprov_2yr==1, 1, 0)

 #Compute C-index (model C)
 cindex_c <-dynpred::cindex(Surv(time_2yr, status_unprov_2yr_dcens)~xb_modc, data=dat.temp)$cindex

 #bootstrap for se of cindex
 B<-100 #(nr of bootstraps)
 set.seed(1234)
 boots_ls_c <- lapply(seq_len(B), function(b) {
 # Resample validation data
 dat.temp_boot <- dat.temp[sample(nrow(dat.temp), replace = TRUE), ]
 # Get cindex on boot validation data
 cindex_c_boot <- dynpred::cindex(Surv(time_2yr, status_unprov_2yr_dcens)~xb_modc, data=dat.temp_boot)$cindex
 cbind.data.frame("cindex"=cindex_c_boot)
 })
 dat.temp_boots_c <-do.call(rbind.data.frame,boots_ls_c)
 cindex_c_se <- sd(dat.temp_boots_c$cindex) #95% CI of the c-statistic is +/-1.96*se;

 ##model D
 #Compute C-index (model C)
 cindex_d <-dynpred::cindex(Surv(time_2yr, status_unprov_2yr_dcens)~xb_modd, data=dat.temp)$cindex

 #bootstrap for se of cindex
 B<-100 #(nr of bootstraps)
 set.seed(1234)
 boots_ls_d <- lapply(seq_len(B), function(b) {
 # Resample validation data
 dat.temp_boot <- dat.temp[sample(nrow(dat.temp), replace = TRUE), ]
 # Get cindex on boot validation data
 cindex_d_boot <- dynpred::cindex(Surv(time_2yr, status_unprov_2yr_dcens)~xb_modd, data=dat.temp_boot)$cindex
 cbind.data.frame("cindex"=cindex_d_boot)
 })
 dat.temp_boots_d <-do.call(rbind.data.frame,boots_ls_d)
 cindex_d_se <- sd(dat.temp_boots_d$cindex) #95% CI of the c-statistic is +/-1.96*se;

 ##Observed/expected ratio
 #Calculate Kaplan-Meier estimate as 'observed'
 obj<-summary(survfit.formula(Surv(time,as.factor(status_unprov==1))~1, data=dat.temp), times=2)
 km<-list(
 "obs"=obj$pstate[, 2],
 "se" =obj$std.err[, 2]
 )
 #Calculate OE model C & D
 OE_C <- km$obs/mean(dat.temp$pred_2jr_modc)
 OE_D <- km$obs/mean(dat.temp$pred_2jr_modd)

 # For the confidence interval we use method proposed in Debray et al. (2017) doi:10.1136/bmj.i6460
 alpha <- 0.05
 OE_C_ll95CI <- exp(log(OE_C) - qnorm(1 - alpha / 2) * km$se / km$obs)
 OE_D_ll95CI <- exp(log(OE_D) - qnorm(1 - alpha / 2) * km$se / km$obs)
 OE_C_se <- (OE_C-OE_C_ll95CI)/1.96 # See main analylsis, se is computed from 95%CI
 OE_D_se <- (OE_D-OE_D_ll95CI)/1.96

 #add results_s1 to dataframe
 result_row<- data.frame(Dataset = i,
 CIndex_C = cindex_c, CIndex_C_se = cindex_c_se,
 CIndex_D = cindex_d, CIndex_D_se = cindex_d_se,
 OE_C = OE_C, OE_C_se = OE_C_se,
 OE_D = OE_D, OE_D_se = OE_D_se)
 results_s1<- rbind(results_s1, result_row)
}
rm(km, boots_ls_c, boots_ls_d, dat.temp, dat.temp_boots_c, dat.temp_boots_d, obj, result_row, alpha, B, cindex_c, cindex_c_se, cindex_d, cindex_d_se, i, OE_C, OE_C_se, OE_C_ll95CI, OE_D, OE_D_se, OE_D_ll95CI)

#### Pool results of sensitivity analysis according to rubin’s rules

#Cindex_C
cindex_c_varb <- var(results_s1$CIndex_C)
cindex_c_varw <- mean(results_s1$CIndex_C_se^2)
cindex_c_vartot <- cindex_c_varw + cindex_c_varb + (cindex_c_varb/m)
cindex_c_se_pooled <- sqrt(cindex_c_vartot)
cindex_c_pooled_s1_summary <- cbind(
 "Cindex" = mean(results_s1$CIndex_C),
 "lower .95" = mean(results_s1$CIndex_C)- 1.96*cindex_c_se_pooled,
 "upper .95" = mean(results_s1$CIndex_C)+ 1.96*cindex_c_se_pooled
)
cindex_c_pooled_s1_summary #Pooled C-statistic model C sensitivity analysis without comp risk
rm(cindex_c_varb, cindex_c_varw, cindex_c_vartot, cindex_c_se_pooled)

#Cindex_D
cindex_d_varb <- var(results_s1$CIndex_D)
cindex_d_varw <- mean(results_s1$CIndex_D_se^2)
cindex_d_vartot <- cindex_d_varw + cindex_d_varb + (cindex_d_varb/m)
cindex_d_se_pooled <- sqrt(cindex_d_vartot)
cindex_d_pooled_s1_summary <- cbind(
 "Cindex" = mean(results_s1$CIndex_D),
 "lower .95" = mean(results_s1$CIndex_D)- 1.96*cindex_d_se_pooled,
 "upper .95" = mean(results_s1$CIndex_D)+ 1.96*cindex_d_se_pooled
)
cindex_d_pooled_s1_summary #Pooled C-statistic model D sensitivity analysis without comp risk
rm(cindex_d_varb, cindex_d_varw, cindex_d_vartot, cindex_d_se_pooled)

#OE_C
OE_C_varb <- var(results_s1$OE_C)
OE_C_varw <- mean(results_s1$OE_C_se^2)
OE_C_vartot <- OE_C_varw + OE_C_varb + (OE_C_varb/m)
OE_C_se_pooled <- sqrt(OE_C_vartot)
OE_C_pooled_s1_summary <- cbind(
 "OE" = mean(results_s1$OE_C),
 "lower .95" = mean(results_s1$OE_C)- 1.96*OE_C_se_pooled,
 "upper .95" = mean(results_s1$OE_C)+ 1.96*OE_C_se_pooled
)
OE_C_pooled_s1_summary #Pooled O/E ratio model C sensitivity analysis without comp risk
rm(OE_C_varb, OE_C_varw, OE_C_vartot, OE_C_se_pooled)

#OE_D
OE_D_varb <- var(results_s1$OE_D)
OE_D_varw <- mean(results_s1$OE_D_se^2)
OE_D_vartot <- OE_D_varw + OE_D_varb + (OE_D_varb/m)
OE_D_se_pooled <- sqrt(OE_D_vartot)
OE_D_pooled_s1_summary <- cbind(
 "OE" = mean(results_s1$OE_D),
 "lower .95" = mean(results_s1$OE_D)- 1.96*OE_D_se_pooled,
 "upper .95" = mean(results_s1$OE_D)+ 1.96*OE_D_se_pooled
)
OE_D_pooled_s1_summary #Pooled O/E ratio model D sensitivity analysis without comp risk
rm(OE_D_varb, OE_D_varw, OE_D_vartot, OE_D_se_pooled)

# Figures

## Figure 2. Calibration plot model C

Code for predictive performance, calibration plots and AUCt plots was adapted from the github provided by *van Geloven et al. Validation of prediction models in the presence of competing risks: a guide through modern methods. BMJ 2022* (PMID: 35609902)

Link to github: <https://github.com/survival-lumc/ValidationCompRisks>

#Preparations
horizon <- 2 #prediction horizon of 2 years
primary_event <- 1 # primary_event = rVTE which is coded as 1

#Calibration curves using LOESS smoothing
#model c
score_modc_2yr_cal <- Score (
 list(df$mean_pred_2jr_modc),
 formula = Hist (time, status_unprov)~1,
 cens.model = "km",
 data=df,
 conf.int = T,
 times = horizon,
 metrics = c("auc"),
 cause = primary_event,
 plots = "calibration" #to save information needed to produce calibration plot
)

# Use pseudo-observations calculated by Score()
pseudos <- data.frame(score_modc_2yr_cal$Calibration$plotframe)
pseudos <- pseudos[order(pseudos$risk), ]

# Use linear loess (weighted local regression with polynomial degree = 1) smoothing
smooth_pseudos <- predict(
 stats::loess(pseudovalue ~ risk, data = pseudos, degree = 1, span = 0.33),
 se = TRUE
)

# prepare histogram of estimated risks for x-axis
spike_bounds <- c(-0.075, 0)
bin_breaks <- seq(0, 0.4, length.out = 100 + 1)
freqs <- table(cut(df$mean_pred_2jr_modc, breaks = bin_breaks))
bins <- bin_breaks[-1]
freqs_valid <- freqs[freqs > 0]
freqs_rescaled <- spike_bounds[1] + (spike_bounds[2] - spike_bounds[1]) *
 (freqs_valid - min(freqs_valid)) / (max(freqs_valid) - min(freqs_valid))

# Produce plot
par(xaxs = "i", yaxs = "i", las = 1)
plot(
 x = pseudos$risk,
 y = pseudos$pseudovalue,
 xlim = c(0, 0.4),
 ylim = c(spike_bounds[1], 0.4),
 yaxt = "n",
 frame.plot = FALSE,
 main = "Calibration plot model C",
 xlab = "Estimated risks",
 ylab = "Observed outcome proportions",
 type = "n"
)
axis(2, seq(0, 0.4, by = 0.1), labels = seq(0, 0.4, by = 0.1))
polygon(
 x = c(pseudos$risk, rev(pseudos$risk)),
 y = c(
 pmax(smooth_pseudos$fit - qt(0.975, smooth_pseudos$df) * smooth_pseudos$se, 0),
 rev(smooth_pseudos$fit + qt(0.975, smooth_pseudos$df) * smooth_pseudos$se)
 ),
 border = FALSE,
 col = "lightgray"
)
abline(a = 0, b = 1, col = "gray")
lines(x = pseudos$risk, y = smooth_pseudos$fit, lwd = 2)
segments(
 x0 = bins[freqs > 0],
 y0 = spike_bounds[1],
 x1 = bins[freqs > 0],
 y1 = freqs_rescaled
)

rm(score_modc_2yr_cal, smooth_pseudos, pseudos, bin_breaks, bins, freqs, freqs_rescaled, freqs_valid, spike_bounds)

## Figure 3 Calibration plot model d

#Calibration curves using LOESS smoothing
score_modd_2yr_cal <- Score (
 list(df$mean_pred_2jr_modd),
 formula = Hist (time, status_unprov)~1,
 cens.model = "km",
 data=df,
 conf.int = T,
 times = horizon,
 metrics = c("auc"),
 cause = primary_event,
 plots = "calibration" #to save information needed to produce calibration plot
)

# Use pseudo-observations calculated by Score()
pseudos <- data.frame(score_modd_2yr_cal$Calibration$plotframe)
pseudos <- pseudos[order(pseudos$risk), ]

# Use linear loess (weighted local regression with polynomial degree = 1) smoothing
smooth_pseudos <- predict(
 stats::loess(pseudovalue ~ risk, data = pseudos, degree = 1, span = 0.33),
 se = TRUE
)

# prepare histogram of estimated risks for x-axis
spike_bounds <- c(-0.075, 0)
bin_breaks <- seq(0, 0.4, length.out = 100 + 1)
freqs <- table(cut(df$mean_pred_2jr_modd, breaks = bin_breaks))
bins <- bin_breaks[-1]
freqs_valid <- freqs[freqs > 0]
freqs_rescaled <- spike_bounds[1] + (spike_bounds[2] - spike_bounds[1]) *
 (freqs_valid - min(freqs_valid)) / (max(freqs_valid) - min(freqs_valid))

# Produce plot
par(xaxs = "i", yaxs = "i", las = 1)
plot(
 x = pseudos$risk,
 y = pseudos$pseudovalue,
 xlim = c(0, 0.4),
 ylim = c(spike_bounds[1], 0.4),
 yaxt = "n",
 frame.plot = FALSE,
 main = "Calibration plot model D",
 xlab = "Estimated risks",
 ylab = "Observed outcome proportions",
 type = "n"
)
axis(2, seq(0, 0.4, by = 0.1), labels = seq(0, 0.4, by = 0.1))
polygon(
 x = c(pseudos$risk, rev(pseudos$risk)),
 y = c(
 pmax(smooth_pseudos$fit - qt(0.975, smooth_pseudos$df) * smooth_pseudos$se, 0),
 rev(smooth_pseudos$fit + qt(0.975, smooth_pseudos$df) * smooth_pseudos$se)
 ),
 border = FALSE,
 col = "lightgray"
)
abline(a = 0, b = 1, col = "gray")
lines(x = pseudos$risk, y = smooth_pseudos$fit, lwd = 2)
segments(
 x0 = bins[freqs > 0],
 y0 = spike_bounds[1],
 x1 = bins[freqs > 0],
 y1 = freqs_rescaled
)

rm(score_modd_2yr_cal, smooth_pseudos, pseudos, bin_breaks, bins, freqs, freqs_rescaled, freqs_valid, spike_bounds)

## Figure 4. Cumulative incidence of rVTE stratified by predicted risk category

#Categorize patients in risk groups
df$risk_group <- cut(df$mean_pred_2jr_modc, breaks = c(0,0.06,0.14,1), labels = c("Low", "Intermediate", "High"))

#Make factor variable for status to use in plot
df$status_unprov_f <- factor(df$status_unprov, levels = c("0","1","2"), labels = c("censored", "Recurrent VTE", "Death"))

#make plot
require(tidycmprsk)
fit_cr_rg <- cuminc(Surv(time, as.factor(status_unprov_f))~risk_group, data = df)
ggcuminc(fit_cr_rg, outcome = c("Recurrent VTE")) +
 labs(x="Time (years)",
 title = "Cumulative incidence of recurrent VTE by riskgroup")+
 add_confidence_interval() +
 add_risktable(risktable_height = 0.25) +
 xlim (0,8) + ylim (0,0.5)

## Figure 5. Histogram of 2-years predicted risks for patients with provoked and unprovoked VTE

#Preparations
labels <-c("Provoked first VTE","Unprovoked first VTE")
names(labels) <- c("0","1")

#create figure
ggplot(df, aes (x=mean_pred_2jr_modc, fill=as.factor(unprovoked), color=as.factor(unprovoked))) +
 geom_histogram(aes(y=..count../sum(..count..)), binwidth=0.01, alpha=0.5, show.legend=F) +
 facet_grid(unprovoked~., labeller = labeller(unprovoked = labels))+
 labs(title = "Histogram of 2-year predicted risks according to model C \nfor patients classified with a provoked and unprovoked first VTE",
 x='2-year predicted risk',
 y= 'Proportion of patients')+
 theme_classic()

# Supplementary materials

## Table S3. Cumulative incidence of recurrent VTE

require(tidycmprsk)
#Unprovoked recurrence
fit_cr <- cuminc(Surv(time, as.factor(status_unprov))~1, data = df)
tbl_cuminc(fit_cr, times = c(1,2,5,10))

#All recurrences
fit_cr_all <- cuminc(Surv(time, as.factor(status))~1, data = df)
tbl_cuminc(fit_cr_all, times = c(1,2,5,10))

## Figure S1. Time dependent AUC model C

#Plot of time dependent AUC of model C over FU period
score_modc<- Score (
 list(df$mean_pred_2jr_modc),
 formula = Hist (time, status_unprov)~1,
 cens.model = "km",
 data=df,
 conf.int = T,
 times = seq(0.1, 8.01, 0.1),
 metrics = c("auc"),
 cause = primary_event,
)

plot(score_modc$times,
 score_modc$AUC$score$AUC,
 type = "l",
 bty = "n",
 xlim = c(0, 8),
 ylim = c(.5, 1),
 lwd = 2,
 xlab = "Time (years)",
 ylab = "AUC",
 lty = 1
)
polygon(c(
 score_modc$times,
 rev(score_modc$times)
),
c(
 score_modc$AUC$score$lower,
 rev(score_modc$AUC$score$upper)
),
col = rgb(160, 160, 160, maxColorValue = 255, alpha = 100),
border = FALSE
)
lines(score_modc$times,
 score_modc$AUC$score$AUC,
 col = "black",
 lwd = 2,
 lty = 2
)
title("Time-dependent AUC model C", adj = 0)

## Figure S2 Time dependent AUC model D

#Plot of time dependent AUC of model D over FU period
score_modd<- Score (
 list(df$mean_pred_2jr_modd),
 formula = Hist (time, status_unprov)~1,
 cens.model = "km",
 data=df,
 conf.int = T,
 times = seq(0.1, 8.01, 0.1),
 metrics = c("auc"),
 cause = primary_event,
)

plot(score_modd$times,
 score_modd$AUC$score$AUC,
 type = "l",
 bty = "n",
 xlim = c(0, 8),
 ylim = c(.5, 1),
 lwd = 2,
 xlab = "Time (years)",
 ylab = "AUC",
 lty = 1
)
polygon(c(
 score_modd$times,
 rev(score_modd$times)
),
c(
 score_modd$AUC$score$lower,
 rev(score_modd$AUC$score$upper)
),
col = rgb(160, 160, 160, maxColorValue = 255, alpha = 100),
border = FALSE
)
lines(score_modd$times,
 score_modd$AUC$score$AUC,
 col = "black",
 lwd = 2,
 lty = 2
)
title("Time-dependent AUC model D", adj = 0)

## Figure S3 Cumulative incidence of the competing risk of death

#make plot
require(tidycmprsk)
fit_cr_rg <- cuminc(Surv(time, as.factor(status_unprov_f))~risk_group, data = df)
ggcuminc(fit_cr_rg, outcome = c("Death")) +
 labs(x="Time (years)",
 title = "Cumulative incidence of the competing risk of death by riskgroup")+
 add_confidence_interval() +
 add_risktable(risktable_height = 0.25) +
 xlim (0,8) + ylim (0,0.5)

## Figure S5 Kaplan-Meier and Aalen-Johansen estimates of observed outcome probabilities

##Compute differences in observed incidence of rVTE/death according to CICR/KM method
#CICR method
summary(survfit(Surv(time, as.factor(status_unprov))~1, data=df), times = c(1,2,5,10))

#KM method
#CI rVTE according to KM (take PR(TRUE))
summary(survfit(Surv(time, as.factor(status_unprov==1))~1, data=df), times = c(1,2,5,10))

#CI death according to KM (take PR(TRUE))
summary(survfit(Surv(time, as.factor(status_unprov==2))~1, data=df), times = c(1,2,5,10))

#CI event-free survival according to KM (take Pr(s0))
summary(survfit(Surv(time, as.factor(status_unprov==1 | status_unprov ==2))~1, data=df), times = c(1,2,5,10))
